# The Impact of Infrastructure on Low‐Income Consumers' Nutritious Diet, Women's Economic Empowerment, and Gender Equality in Low‐ and Middle‐Income Countries: An Evidence and Gap Map

**DOI:** 10.1002/cl2.70050

**Published:** 2025-07-18

**Authors:** Clarice Panyin Nyan, Gloria A. Odei Obeng‐Amoako, Joseph Clottey, Sheila Agyemang Oppong, Charles Yaw Okyere, Takyiwaa Manuh, Solomon Zena Walelign, David Sarfo Ameyaw

**Affiliations:** ^1^ International Centre for Evaluation and Development, Tema Accra Ghana

## Abstract

Physical infrastructure, such as market centers and roads, can foster women's economic empowerment and gender equality and mitigate adverse effects of seasonality on availability and prices of nutritious foods. The lack of infrastructure is therefore a major challenge for agricultural development in Sub‐Saharan Africa and South Asia—the regional focus of this study. It threatens food and nutrition security, depriving low‐income consumers' access to healthy, affordable food and quality nutrition. Interestingly, previous studies show that physical infrastructure promotes inclusive growth and maximizes positive impacts such as improved well‐being and sustainable development, and can contribute to the empowerment of women and girls. When infrastructural investments are planned, delivered, and managed using nutrition‐sensitive, gender‐inclusive, and responsive approaches, it can help to address barriers that impede access to nutritious diets, nutrition security, and structural inequities militating against women and girls at the household and market levels. Hence, investments in physical infrastructure could be a useful pathway for meeting various Sustainable Development Goals (SDGs 1–No Poverty, 2–Zero Hunger, 3–Good Health and Wellbeing, 5–Gender Equality, 6–Clean water and sanitation, 7–Affordable and Clean Energy, and 8–Decent Work and Economic Growth). However, few studies have examined the evidence and gaps on infrastructure's impact on nutritious diet, women's economic empowerment, and gender equality in low‐ and middle‐income countries (LMICs) in Sub‐Saharan Africa and South Asia. Evidence and gap maps are useful tools for promoting evidence‐informed decision‐making by making evidence and research gaps accessible to policymakers, development practitioners, and researchers. This EGM was conducted in the consultations with stakeholders. This study seeks to identify, map, and provide an overview of the existing evidence and gaps on the impact of physical infrastructure on nutritious diets, women's economic empowerment, and gender equality among low‐income consumers in LMICs in sub‐Saharan Africa and South Asia regions. A standardized search strategy was adapted for searching published and unpublished studies in 3 academic databases, 33 institutional websites, Google, Google Scholar, 3 existing EGMs, and 8 registries of randomized control trials and pre‐analysis plans from June 2022 to September 2022. Additional papers were identified through OpenAlex in EPPI‐Reviewer. We supplemented the database searches by conducting hand searches and backward citation searches in identified reviews for relevant studies. We also contacted five prominent authors in the literature for relevant completed and on‐going studies for the EGM. The selection criteria adapted the PICOS (population, intervention, comparison, outcomes, and study design) approach. The intervention was defined as those related to establishing or upgrading physical infrastructure for the agricultural sector and local economic development, such as production, post‐production, distribution, and information. Furthermore, the outcomes were nutritious diets, women's economic empowerment, and gender equality. This EGM does not specify a comparison group. Two other eligibility criteria for including studies were publication in the Year 2000 and onwards, and those studies written in the English language. A total of 17,102 studies were uploaded and screened in EPPI‐Reviewer data management software for titles and abstracts. About 969 studies were screened for full‐text, and 342 eligible studies were included in the map based on a pre‐defined code. The unit of analysis was a study. Therefore, each item presented in the EGM is a study. Studies reporting multiple interventions, outcomes, or study designs were coded multiple times per the appropriate coding category, but counted as one entry in the EGM. All outliers and out‐of‐range frequency values of assigned codes were identified and cleaned. Data was analyzed using descriptive statistics in Microsoft Excel and STATA version 16. The EGM was generated using EPPI‐Mapper. A total of 342 studies (337 completed and 5 ongoing studies) from 54 countries across the sub‐Saharan Africa and South Asia regions were included in the EGM. The EGM shows a steady growth in evidence over the last two decades. Most of the studies were impact evaluations (*n* = 178), followed by summative evaluations (*n* = 101). Non‐experimental evaluation (*n* = 255) was the most common study design employed, followed by qualitative studies (*n* = 94), systematic review (*n* = 9), and scoping and other reviews (*n* = 48). This EGM did not find any studies using randomized controlled trials. The few systematic reviews included in the EGM had no accompanying meta‐analysis. The most studied regions were Eastern Africa (*n* = 133), followed by West Africa (*n* = 100) and South Asia (*n* = 93). Production infrastructure (*n* = 202) had most of the evidence, compared with post‐production infrastructure (*n* = 125), distribution infrastructure (*n* = 41), and information infrastructure (*n* = 2). Nutritious diets outcomes (*n* = 274) were the most reported indicators, compared with women's economic empowerment (*n* = 89) and gender equality (*n* = 53) outcomes. The aggregate map showed that production infrastructure and nutritious diets had the most cluster of evidence (*n* = 188) and this suggest a potential area for future evidence synthesis. This EGM presents evidence and research gaps around infrastructural interventions related to nutritious diets, women's economic empowerment, and gender equality, with specific references to the continents of Sub‐Saharan Africa and South Asia. Most of the evidence is based on non‐experimental impact evaluations, and we could not find any randomized controlled trials—a critical gap for future research. The majority of evidence was gathered in Eastern Africa, whereas Central Africa was the least documented. The most studied intervention was irrigation, and more evidence was found on nutritious diets than on women's empowerment and gender equality. This is important at the academic level and at the policy level to assist resource allocation and to support evidence‐based policy tools such as systematic reviews and policy briefs.

**Abbreviations**
ANH: Agriculture, Nutrition & Health
BMGF: Bill & Melinda Gates Foundation
EGM: evidence and gap map
GNI: gross national income
ICED: International Center for Evaluation and Development
LMICs: low‐ and middle‐income countries
PICOS: population, intervention, comparison, outcome, study design
SA: South Asia
SDGs: Sustainable Development Goals
SSA: Sub‐Saharan Africa
ToC: Theory of Change
UNOPS: United Nations Office for Project Services
WEAI: Women's Empowerment in Agriculture Index
WEE: Women's Economic Empowerment
WHO: World Health Organization
WWGS: What Works Global Summit

## 1 Plain Language Summary

### 1.1 The Evidence for the Impact of Infrastructure on Nutritious Diets, Women's Economic Empowerment, and Gender Equality Is Unevenly Distributed and Under‐Reviewed

This evidence and gap map (EGM) shows the available evidence and gaps in the evidence on the impact of physical infrastructure for agriculture, food production, and marketing on nutritious diets, women's economic empowerment, and gender equality in low‐ and middle‐income countries (LMICs) in sub‐Saharan Africa (SSA) and South Asia (SA) regions. The evidence is not evenly distributed by geography and outcome domains. There is significant evidence on food production infrastructure and nutritious diets, but little evidence on the impact of infrastructure on women's economic empowerment and gender equality. Much of the evidence in the EGM is non‐experimental evaluation.

#### 1.1.1 What Is the EGM About?

Emerging evidence shows that infrastructure for agriculture, food production, and marketing plays a vital role in supporting women's economic empowerment, gender equality, and food security by reducing seasonal food price fluctuations and improving access to nutritious food in LMICs in SSA. However, these regions also suffer from the largest infrastructure deficits globally. Decision‐makers lack adequate evidence to guide infrastructure investments, scale‐ups, renovations, and advocacy efforts.

This EGM presents available evidence and gaps on the impact of physical infrastructure for agriculture, food production, and marketing on nutritious diets, women's economic empowerment, and gender equality among low‐income consumers (LICs) in LMICs in SSA and SA regions.

#### 1.1.2 What Studies Are Included?

The EGM includes 342 studies (337 completed and 5 ongoing), covering impact, process, formative, and summative evaluations, as well as qualitative and non‐experimental studies, systematic reviews, scoping reviews, and other reviews published over the last two decades.

Infrastructure is defined by four categories:

1. Production infrastructure encompasses facilities for agricultural production (e.g., irrigation systems, on‐farm energy sources);
2. Post‐production infrastructure includes facilities used for either (i) storing, processing, and marketing agricultural products and (ii) ensuring the supply of healthy foods in safe environments (e.g., cold rooms, processing facilities, marketing facilities);
3. Distribution infrastructure includes facilities for transporting agricultural inputs and products (e.g., roads, railways);
4. Information infrastructure consists of facilities for the dissemination of information to producers and consumers of agricultural products (e.g., radio stations and telecommunication).

#### 1.1.3 What Are the Main Findings of This EGM?

The majority of studies focus on Eastern Africa, West Africa, and SA, with Central Africa being the least studied. Ethiopia is the most studied country, likely due to interest in irrigation and nutrition outcomes. Generally, studies on infrastructural interventions and outcomes are unevenly distributed.

Production infrastructure, particularly irrigation systems and water wells, is the most studied (202 out of 370 studies), followed by post‐production infrastructure (125 out of 370 studies) and distribution infrastructure (41 out of 370 studies). The information infrastructure received less research interest. There are more studies on nutritious diet outcomes (274 out of 416 studies) than on women's economic empowerment (89 out of 416 studies) and gender equality (53 out of 416 studies).

Most nutritious diet studies focus on production infrastructure (188 studies). In contrast, very few studies explore how market facilities, storage, or food processing facilities affect women's economic empowerment or gender equality.

Among studies on women's economic empowerment, many assess how off‐farm energy and power supply influence women's time use, access to productive resources, and control over income. Given the critical role women play in food systems and economic development, more primary studies are urgently needed to inform policy and interventions. Distribution infrastructure, such as roads, remains under‐researched in relation to all outcomes.

Most included studies are primary research. There are a few systematic reviews (*n* = 9) without meta‐analysis, and scoping and other reviews (*n* = 48). Only nine systematic reviews (none with meta‐analysis) and 48 other reviews were identified. There are no effectiveness studies, highlighting a critical gap for rigorous evaluations to guide infrastructure policy.

Overall, the EGM compiles 342 studies from 54 countries across SSA and SA, offering an interactive, easy‐to‐navigate platform for users.

#### 1.1.4 What Do the Findings of This Map Mean?

International Center for Evaluation and Development (ICED) plans to use this EGM to promote evidence‐based decision‐making products such as evidence portals, summaries of evidence for specific interventions and outcomes, policy briefs, and checklists for practitioners and policymakers. ICED will use these products to advocate for further primary studies and rigorous systematic reviews in the under‐researched thematic and geographic areas.

In addition, the EGM will support the development of a community of practice focused on infrastructure for agriculture, food production, and marketing. It will also contribute to capacity‐building efforts, promoting the use of systematic reviews, meta‐analyses, and experimental methods in research.

#### 1.1.5 How Up‐to‐Date Is This EGM?

The authors searched for and included studies published from 2000 to March 2023.

## 2 Background

Governments of developing countries, particularly in SSA and SA, have implemented various physical infrastructural projects as they are essential for development and achieving the Sustainable Development Goals (SDGs). Several researchers and practitioners across diverse fields of study have highlighted that access to infrastructure is important for improving nutritional outcomes, women's economic empowerment, and gender equality (Morgan et al. 2020; Mohun and Biswas 2016). This implies that the lack of well‐functioning infrastructure has a negative impact on these outcomes, particularly among LICs (WHO 2022). Hence, investment in infrastructure is essential in ensuring access to nutritious diets, improving women's economic empowerment, and advancing gender equality, especially in the ongoing discourse on sustainable food systems (Njuki et al. 2023). However, investments in physical infrastructure, particularly in developing countries, tend to overlook important gender and nutrition outcomes, such as the consumption of nutritious diets, women's economic empowerment, and gender equality.

Seasonality has considerable negative effects on food and nutrition security among LICs in SSA and SA, affecting the availability and price of foods year‐round (Bai et al. 2020; Gilbert et al. 2017). Seasonality is a key determinant of food availability, especially perishable and costly foods like fruits and vegetables. The effects of seasonality on food affordability, accessibility, and availability are manifested through complex causal pathways (Bai et al. 2020). Emerging evidence shows that physical infrastructure such as roads, market centers, storage, and food processing facilities enhance access to affordable and safe, nutritious food year‐round (Shively and Thapa 2017). Households residing closer to market centers are more likely to consume more nutritious diets than those who reside farther away from market centers (Usman and Haile 2019).

Further, previous studies also show that physical infrastructure could foster women's economic empowerment and gender equality and thereby improve the livelihoods of women and girls (Morgan et al. 2020). Even when physical infrastructure exists, it is often not designed to be inclusive of gender‐specific needs. However, when infrastructure is not designed inclusively, it may limit access to resources and opportunities for all groups, with evidence showing that women and girls are often more adversely affected due to existing gender inequalities (Morgan et al. 2020). Hence, gender responsiveness must guide the design and uptake of future infrastructure, especially those related to the agri‐food systems (Mohun and Biswas 2016; Morgan et al. 2020).

Furthermore, literature shows that infrastructure is a game‐changer for development and enhances women's economic opportunities (Mohun and Biswas 2016). For instance, investment in well‐functioning infrastructure such as market centers and roads, could foster gender‐inclusive development and mitigate the adverse effects of seasonality on the availability and price of nutritious foods (Shively and Thapa 2017), and reduce child undernutrition in developing countries (Shively 2017). Hence, investments in physical infrastructure could be a useful pathway for meeting various SDGs (SDGs 1–No Poverty, 2–Zero Hunger, 3–Good Health and Wellbeing, 5–Gender Equality, 6–Clean water and sanitation, 7–Affordable and Clean Energy, and 8–Decent Work and Economic Growth) (Shively 2017; Shively and Thapa 2017; Thapa and Shively 2018; World Bank 2020), especially if the design, placement, and construction of infrastructure are guided by gender inclusivity and nutrition sensitivity principles (Mohun and Biswas 2016; Morgan et al. 2020). However, there is a paucity of evidence on the impact of infrastructure on nutrition and gender outcomes to inform decision‐making in low‐ and middle‐income (LMICs) countries. Few studies have examined infrastructure's impact on nutritious diets, women's economic empowerment, and gender equality in LMICs (Belete and Melak 2020; Domènech and Ringler 2013). This study seeks to address this gap in the literature by identifying, mapping, and providing an overview of the existing evidence and gaps on the impact of different types of physical infrastructure on nutritious diets, women's economic empowerment, and gender equality among LICs in LMICs in SSA and SA.

### 2.1 The Intervention

The intervention for this EGM is *physical infrastructure*, broadly defined as *tangible, physical assets, such as roads, market centers, storage facilities, and food processing units, that are needed for a society or enterprise to run smoothly* (Oxford Press 2018; World Bank 2020). The EGM focuses on types of physical infrastructure that are relevant for agriculture, food production, and marketing, and thus local development. Based on extant literature, this EGM categorizes the interventions into four types of infrastructures: production, post‐production, distribution, and information (Daccache et al. 2013; Gajigo and Lukoma 2011).

- Production infrastructure encompasses facilities that are used in agricultural production or any other facilities that are in place to enhance agricultural productivity. Examples of such infrastructure include irrigation systems, water wells, reservoirs, greenhouses, agrivoltaics, and on‐farm energy sources.
- Post‐production infrastructure includes facilities that are used either to (i) store, process, and market agricultural products and (ii) ensure the supply of healthy foods in safe environments. Examples of post‐production infrastructure include storage facilities (warehouses and sheds, cold rooms, pack houses), processing facilities (grain mills), marketing facilities (spaces, stalls, and lockups, and others (slaughterhouses and landing sites).
- Distribution infrastructure includes facilities that are used for transporting agricultural inputs and products. Examples of distribution infrastructure include roads, railways, and bridges.
- Information infrastructure consists of facilities that support the dissemination of information to producers and consumers of agricultural products. Examples of information
- Infrastructure includes information centers, radio stations, and telecommunication masts.

### 2.2 Why It Is Important to Develop the EGM

This EGM organizes a body of evidence on the effects of infrastructure on nutritious diets, women's economic empowerment, and gender equality, to inform investment decision‐making. The EGM is a useful public good to researchers, funders, and policymakers to guide and pinpoint where to allocate resources for further studies, systematic reviews, or new interventions, ensuring that future investments are evidence‐informed and targeted at addressing the most critical gaps in knowledge. The EGM would help researchers and experts to prioritize evidence generation, and for policymakers and other stakeholders to prioritize research funding and infrastructural investments. Finally, this EGM makes vital evidence easily accessible to policymakers and researchers who hitherto would be unaware of existing evidence and gaps on the topic under consideration.

### 2.3 Existing EGMs and Relevant Systematic Reviews

Four earlier identified EGMs (Moore et al. 2021; Leveraging Evidence for Access and Development 2021; Sparling et al. 2022; Malhotra et al. 2021) addressed some of the aspects of the interventions and outcomes in the current EGM. First, the EGM on “the effects of food systems interventions on food security and nutrition outcomes in low‐ and middle‐income countries” focused on studies related to food value chains, food environment, and consumer behavior (Moore et al. 2021). The second EGM focused on “gender in agriculture and food systems.” This EGM aimed to catalog the evidence on the role of gender in improving food and nutrition security in LMICs of Asia, Africa, South America, Middle East, and North Africa (MENA) (LEAD 2021). It highlighted the gender issues in agriculture and food systems, but did not study infrastructure as an intervention. A third EGM aimed to “systematically identify and map analytical studies associating food security and nutrition with mental health.” The study did not mention the role of infrastructure as an intervention in the EGM. The fourth EGM was on “studies of the effectiveness of transport sector interventions in low‐ and middle‐income countries: An evidence and gap map” (Malhotra et al. 2021). Though transport infrastructure was the main intervention of interest, the EGM did not relate transport infrastructure, such as roads and railways, to nutritious diets, women's economic empowerment, and gender quality. Similarly, few existing systematic reviews studied the impact of some types of infrastructure on food security outcomes (Adu et al. 2018; Aziz et al. 2022; Daccache et al. 2013; Domènech 2015; Feron 2016; Jeuland et al. 2021; Kamwamba‐Mtethiwa et al. 2016).

To the best of our knowledge, the current EGM is a novelty, as it provides comprehensive evidence and gaps on typologies of infrastructure that are essential for agricultural development. It also provides an overview of the effect of physical infrastructure on nutritious diets, women's economic empowerment, and gender equality in LMICs across SSA and SA regions. This EGM included a wide range of methodological approaches, including experimental, non‐experimental (quasi‐experimental, regression‐based, and descriptive studies), qualitative studies, and reviews (systematic, scoping, and other literature reviews). This ensured that the EGM captured existing evidence on the topic comprehensively. This EGM provides evidence needed to inform decision‐making on infrastructural investments towards sustainable economic growth and development in LMICs across SSA and the SA regions.

### 2.4 Objectives

This EGM aims to identify, map, and provide an overview of the existing evidence and gaps on the impact of different types of physical infrastructure on LICs' nutritious diets, women's economic empowerment, and gender equality in LMICs. The specific objectives of the EGM are:

i. Identify clusters of evidence that offer opportunities for evidence synthesis to inform future policy and research.
ii. Identify gaps in evidence where new studies, research, and evaluations are needed.

These evidence clusters can inform policymakers about appropriate strategies and where to intervene for maximum impact. EGM would outline best practices and successful interventions. This would help prioritize funding to support research in areas of evidenced gaps, hence efficient usage of resources, informed decisions on policy.

## 3 Methods

### 3.1 EGM: Definition and Purpose

An EGM is defined as *a systematic visual presentation of the availability of relevant evidence of effects for a particular policy domain* (Saran and White 2018). We used a systematic search protocol taking into consideration the PICOS to identify the evidence. Unlike systematic reviews, this EGM does not synthesize the findings reported by the studies but reports on prevalence and characteristics of studies (Potter 2010). However, a descriptive report that summarizes the evidence and gaps is presented along with the map for use by relevant stakeholders such as researchers, research commissioners, policymakers, and practitioners (Saran and White 2018). This EGM is freely accessible to potential users as a public good. It provides clusters of evidence on the linkages between physical infrastructure and nutritious diets, women's economic empowerment, and/or gender equality for future evidence and gaps synthesis to inform primary research and evaluations.

The EGM will provide clear visual presentations of available evidence and research gaps, to facilitate informed decision‐making, enable policymakers and practitioners to quickly understand the breadth of evidence on specific topics. It would guide researchers in areas needing further investigation and support funding agencies in resource allocation. The EGM will also facilitate collaboration between cross‐disciplinary teams and stakeholders; they allow knowledge translation in a way that reduces complexity, and they are useful for educational purposes. But they should not replace detailed reviews and should be used for strategic planning and prioritization of research, nor should any efforts be made toward making causal conclusions based on sparse data.

#### 3.1.1 Framework Development and Scope

The framework development followed several steps. First, all the researchers engaged in the study were trained on how to develop EGMs using the relevant software: EPPI‐Reviewer and EPPI‐Mapper. The team identified the PICOS of the study based on which the title registration form was completed and submitted for registration at the Campbell Collaboration Review. Upon acceptance of the title registration of the EGM, a standard protocol for an EGM as prescribed by the Campbell Collaboration was drafted and submitted to the Campbell Collaboration Review Journal for publication. The protocol was used to systematically guide the production of the EGM. The team then identified relevant academic databases and organizational websites to undertake the search for relevant materials using the designed coding and screening tools. Howard White and Suchi Malhotra again provided training in May 2022.

Campbell Collaboration team was engaged throughout the entire process of the EGM generation. They provided written and oral feedback on the PICOS, the title registration, protocol, and the EGM. Howard White and Ashrita Saran provided weekly advice to the team and commented on different documents: the PICOS, the title registration, protocol, the EGM, and the screening and coding tools. Suchi Malhotra also provided support on EPPI‐Reviewer to the team. Rodney Malesi served as an information specialist and conducted the searches on academic databases and on Google Scholar under the supervision of Ashrita Saran. The preliminary EGM was presented at three conferences: Agriculture, Nutrition and Health (ANH) academy, Evidence to Action (E2A), and What Works Global Summit (WWGS) in 2022. In March 2023, an international webinar was held to present the findings of the pre‐final EGM to stakeholders for their feedback. Lastly, in March 2023, the preliminary EGM was uploaded on ICED's website for public viewing and feedback.

### 3.2 Stakeholder Engagement

Development practitioners, academicians, and policymakers familiar with the subject matter on the scope, design, and production of the EGM were consulted. With the support of Campbell Collaboration, the conceptualization, the design and the production were made. The views and inputs of stakeholders were consulted at every stage of the EGM development to ensure the EGM is fit for purpose and useful to inform future research, policy, program, and funding decision‐making on infrastructure, nutritious diet, women's economic empowerment, and gender equality. The final EGM is widely disseminated amongst relevant organizations, institutions, and networks for use and application.

### 3.3 Evidence‐Based Theory of Change (EBToC)

**Figure 1:**
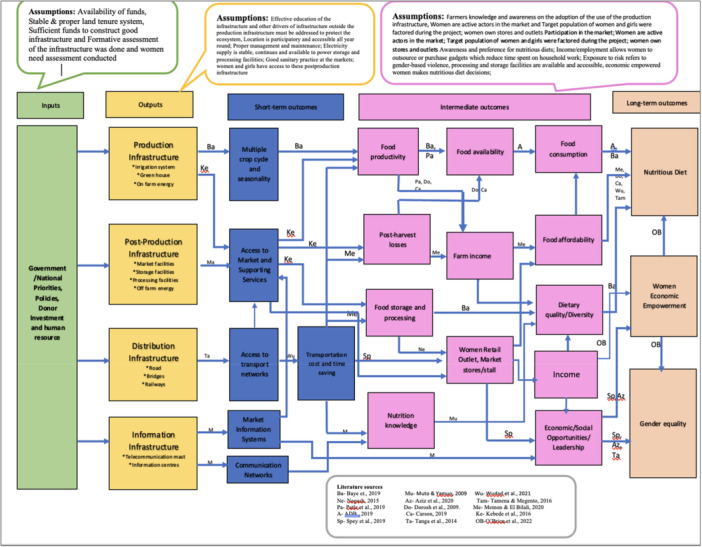
Evidence‐Based Theory of Change for infrastructural interventions on nutritious diets, women's empowerment, and gender equality.

The EBToC presented in Supporting Information (Figure 1) illustrates the pathways through which infrastructural interventions in production, post‐production, distribution, and information systems lead to desired outcomes such as nutritious diets, women's economic empowerment, and gender equality. This EBToC is based on studies that establish links between infrastructure and outcomes related to nutrition and gender. The framework demonstrates the mechanisms by which infrastructure contributes to short‐term, intermediate, and long‐term outcomes. However, while short‐term outcomes are an integral part of the theoretical framework, the current evidence base did not provide sufficient data to include these in the descriptive analysis.

The ToC identifies four pathways: production, food‐loss reduction, communication, and transport. The production pathway highlights how production infrastructure, such as irrigation, impacts agricultural productivity, leading to increased food availability at the farm level and ultimately improving food consumption and nutrition. Access to markets and supporting services is another pathway facilitated by production infrastructure, further enhancing nutritious diets (Figure 1).

Research by Kebede et al. (2021) confirms that production infrastructure, including irrigation, improves farmers' access to agricultural inputs, thereby enhancing productivity and food availability. Power supply and women's empowerment are linked through complex socio‐political and economic factors, as noted by Clancy et al. (2007). The provision of energy services, as highlighted by Winther et al. (2016), positively affects women's well‐being, including health, education, and income opportunities.

Distribution infrastructure plays a crucial role in improving access to transportation networks, reducing costs and time, establishing retail outlets, and contributing to food storage, processing, and reduced post‐harvest losses. Dorosh et al. (2010) and Carson et al. (2022) highlight how distribution infrastructure empowers women and enables households to consume nutritious foods.

The communication pathway demonstrates how information infrastructure, such as telecommunication systems, contributes to long‐term outcomes related to nutritious diets, women's economic empowerment, and gender equality. Post‐production infrastructure, similar to production infrastructure, results in improved access to markets and supporting services, ultimately leading to enhanced food consumption and nutrition.

The robustness of this framework relies on key assumptions, including the availability of funds from government or donor agencies for infrastructure construction and the existence of formative evaluations of the infrastructure projects. Additionally, it is assumed that participants have received adequate education and training on utilizing the infrastructure.

### 3.4 Dimensions

The major dimensions of this EGM are interventions, outcomes, evaluation types, and study designs. The interventions and the outcomes are respectively presented in rows and columns, resulting in a matrix, where each cell shows the number of studies in all possible combinations of the intervention and outcome categories. The evaluation types and study designs are presented as a segment of the number of studies in each cell. The outcome dimension includes nutritious diets, women's economic empowerment, and gender equality, and some sub‐domains under each of the outcomes. The intervention dimension includes four major physical infrastructure (production, post‐production, distribution, and information) and some sub‐categories under each physical infrastructure.

We allow for multiple coding for studies that reported evidence on more dimensions of the EGM. The EGM included studies from any study designs, including those that are descriptive and qualitative in nature, and different types of evaluations, including formative and process evaluation studies.

### 3.5 Types of Study Design

This EGM targeted all studies that aimed to provide evidence on the impact of physical infrastructure on nutritious diet, women's economic empowerment, and gender equality in LMICs. Hence, no study was excluded based on study design, provided it used primary or empirical data. The EGM also included systematic reviews and reviews with less rigorous literature search methodology compared with systematic reviews like scoping reviews and other literature reviews on the topics of interest. Specifically, studies employing the following study designs were included:

- *Experimental analysis:* This included studies that made use of random assignment of subjects into treatment and/or control. All studies that used randomized evaluation techniques such as randomized controlled trials (RCTs) were coded under this category. However, as previously stated, this study did not find papers using RCTs on the topic of interest.
- *Non‐experimental analysis:* This included studies employing other quantitative approaches such as quasi‐experimental techniques, regression methods, and descriptive statistics. Specific techniques considered under this category included difference‐in‐difference, regression discontinuity design, matching (e.g., propensity score matching, etc.), synthetic controls, endogenous switching regression, panel data analysis techniques (fixed effects, random effects, correlated random effects, etc.), ordinary least squares, instrumental variables (IV), two‐stage least squares, among others.
- *Reviews:* All kinds of reviews such as systematic with or without meta‐analysis, literature and scoping reviews were included in the EGM.
- *Qualitative analysis:* The EGM included studies that employed qualitative designs such as narrative and exploratory approaches and data collection methods such as focus group discussion and interviews to examine the relationship between physical infrastructure and outcomes of interest.

### 3.6 Types of Evaluation

Only studies with the following types of evaluation were included in the EGM: impact evaluation, process evaluation, formative evaluation, and summative evaluation (CDC n.d.).

- Impact evaluation: This type of evaluation assesses the effectiveness (causal inferences) of a program or intervention in attaining its overall goal. For instance, a non‐governmental organization assesses the impact of a nutrition program aimed at reducing malnutrition rates in rural communities over 5 years. The evaluation measures changes in malnutrition rates, school attendance, and community health indicators among children before and after program implementation.
- Process evaluation: This evaluation type ascertains if the intended activities of a program have been executed. For instance, a government agency conducts a process evaluation for a new public health campaign on smoking cessation. This evaluation assesses the implementation process, including resource allocation, staff training, outreach methods, and how closely the actual activities align with the program design.
- Formative evaluation: This type of evaluation is usually done when a new program is being developed or an existing one is being altered, and ensures that such programs are suitable, acceptable and ultimately achievable. Before launching a community‐based recycling program, a city conducts a formative evaluation to gather input from residents on recycling habits and preferences. Feedback collected helps refine the program's design, targeting areas such as accessibility, educational outreach, and collection frequency.
- Summative evaluation: This type of evaluation assesses the attribution of a program or intervention in attaining an outcome. A school district completes a summative evaluation of a year‐long digital literacy program in middle schools, assessing final student outcomes like digital skills proficiency, test scores, and engagement levels. This evaluation is conducted at the end of the program to judge its overall effectiveness.

### 3.7 Types of Interventions

We considered both newly established as well as upgrading of existing infrastructure. Focusing on infrastructure that were relevant for agriculture, food production, marketing, and thus local development, we identified and focused on four types of infrastructure: production, distribution, post‐production, and information (see Table 1 for domains and sub‐domains of infrastructure and their definitions). The study did not focus on physical infrastructure, which had multi‐components.

**Table 1:** Categories, sub‐categories, and intervention examples (infrastructure).

Categories of infrastructure	Definition	Subcategory/examples
Production infrastructure	Facilities that are used in agricultural production or any other facilities that are in place to enhance agricultural productivity.	Irrigation systems, water wells, Greenhouse, on‐farm energy and power supply (solar, wind, water, etc.)
Post‐production infrastructure	Facilities that are used for storing, processing, and marketing products as well as facilities that are used to ensure the supply of healthy foods in safe environments.	− Storage (warehouses and sheds, cold rooms, pack houses) − Processing (grain mills) − Market place (spaces, stalls, and lockups, provided with sanitary facilities (toilets) and childcare centers) − Off‐farm energy and power supply (solar, wind, water) − Others (slaughterhouses, landing sites)
Distribution infrastructure	Facilities that are used for transporting inputs and products to ensure that LICs, including farmers, have access to markets and increased access to nutritious and diversified foods.	− Roads, − Railways, − Bridges
Information infrastructure	Facilities used to support the dissemination of information on good agricultural practices, nutritional knowledge, child feeding practices, weather information, markets, and credit, etc.	Information centers, radio stations, telecommunication masts to facilitate and enhance communication

The EGM focused on *physical presence* of the infrastructure, so studies focusing on the use or services provided by the infrastructure were excluded from the EGM.

### 3.8 Types of Population

The population of interest for this EGM was LICs in lLMICs across SSA and SA regions. Following Darley and Johnson (1985), we defined LICs as “individuals whose financial resources or income results in them being unable to obtain the goods and services needed for an ‘adequate’ and ‘socially acceptable’ standard of living.” Similarly, following World Bank (2020), we defined LMICs as countries with per capita gross national income (GNI) below US$12,695 (World Bank 2020). This definition encompasses lower income countries (GNI less than US$1,046), lower middle‐income countries (GNI ranging from US$1,046 to 4,095), and upper middle‐income countries (GNI per capita between US$4,096 and US$12,695). Studies that exclusively focus on middle‐ and/or high‐income consumers were excluded from the EGM. Studies whose study region fell outside of SSA and SA were also excluded.

### 3.9 Types of Outcome Measures

Studies focusing on one or more of the three outcomes (i.e., nutritious diets, women's economic empowerment, and gender equality) were included in the EGM. The outcomes were represented by a number of sub‐domains (see Table 2 for the definition and indicators of the subdomains).

**Table 2:** Categories and sub‐domains of outcomes with examples.

Outcome categories	Sub‐outcome categories	Examples of indicators/measures
Nutritious^a^ diets	Food availability: farm level	Food production or yield (e.g., quantity of food produced per area [kg/hectare])
	Food availability: market level food	Availability of nutritious foods (fruits, vegetables, dairy, eggs, meat, fish, legumes, nuts) (e.g., Specific metrics could be market volumes sold or transported or aggregated, number of market stands selling these items, etc.), Market Level Dietary Score (MLDS): Number of foods or food groups available in local markets at a given point in time
	Food accessibility: affordability	Volatility of food prices, change in income, household food expenditure, household food expenditure, Food Insecurity Experience Scale (FIES), Household Food Insecurity Access Scale (HFIAS), Household Hunger Scale (HHS)
	Food accessibility (physical)	Presence of food market, proximity, physical presence of food, food vendors
	Diet quality^b^ (nutrient adequacy) individual dietary diversity	Individual dietary diversity scores (DDS), Minimum Acceptable Diet (MAD) for infant/child (6–23 months), Minimum Dietary Diversity (MDD) for infant/child (6–23 months), Minimum Dietary Diversity for Women between 15 and 49 years (MDD‐W), Mean adequacy ratio (MAR): measures an individual's intake of nutrient, Food variety score, household caloric intake
	Diet quality (nutrient adequacy) household dietary diversity	Household dietary diversity score, Food variety score, Food groups consumed, Food Consumption Score (FCS), and dietary energy per capita caloric (kcal)
	Socioeconomic and cultural dimensions of foods	Women empowerment in nutrition index (WENI), food preferences. WENI is a nutrition‐centered metric of empowerment that can be used to measure, track, and identify barriers to nutritional empowerment
Women's economic empowerment^c^	− Agricultural production	− Input in production decisions − Autonomy in production
	− Access and control over productive resources	− Ownership of assets − Purchase, sale, or transfer of assets − Access to and decisions about credit
	− Income	− Control over the use of income
	− Time allocation	− Workload, Leisure
	− Leadership	− Group Member, Speaking in Public
	*Women Empowerment in Agriculture Index* (WEAI)	
	Gender Parity Index (GPI)^d^	Women's achievements in the five domains (5DE)^e^, relative to the men in their households^f^
Gender equality^g^	Economic opportunities and outcomes	− Women and men have equal opportunities in agricultural production systems, value chains, markets, resources (GAGP)^h^, and entrepreneurship
	Social outcomes	− Discriminatory and unequal social, cultural, and gender norms change to enable women and men to participate equally in household and community institutions
	Leadership, agency and collective action	− Women's agency, leadership, and decision‐making recognized and affirmed in the household and community − Women can engage in collective action to protect their interests
	Reduced exposure to risk	− Gender‐based violence (Cf. Pro‐WEAI + MI)‐ legislation and institutions exist to protect against GBV, and women are empowered to take action, leading to low and reduced rates of GBV^i^

^a^ Nutritious Diet: A nutritious diet provides essential nutrients in appropriate amounts to support physical and mental health, development, and well‐being. It emphasizes a balance of proteins, carbohydrates, fats, vitamins, and minerals from diverse food sources, aligning with current dietary guidelines for optimal health. Nutritious diets are vital for preventing malnutrition and managing health risks, particularly in addressing global issues like obesity, diabetes, and undernutrition (FAO 2022; WHO 2022).

^b^ Dietary diversity represents qualitative measures of individual or household food consumption that reflects access to a variety of food groups and used as a proxy for nutrient adequacy of the diet of individuals (INDDEX Project 2019; Kennedy et al. 2011). We usually count nine food groups listed as follow: (1) cereals, (2) starchy roots, (3) legumes, (4) vegetables and fruits, (5) sugars, preserves, and syrups, (6) meat, fish, and eggs, (7) milk and milk products, (8) fats and oils, and (9) beverages.

^c^ This is measured using the Women's Empowerment in Agriculture Index (WEAI) (Alkire et al. 2013). WEAI is an aggregate index and is composed of two sub‐indexes – the five domains of empowerment (5DE), and the gender parity index (GPI). 5 Domains of empowerment (5DE), specified in the sub‐outcomes (Alkire et al. 2013). The overall WEAI is a weighted average of 5DE and GPI, with weights 0.9 and 0.1, respectively (Malapit and Quisumbing 2015).

^d^ The GPI measures women's achievements in the 5 domains compared to the men in their households. All these indexes have values ranging from 0 to 1, where higher values reflect greater empowerment. Households are classified as having gender parity if either the woman is empowered (her empowerment score is 80% or higher) or her score is greater than or equal to the empowerment score of the male decision‐maker in her household. It is only calculated for dual‐headed households.

^e^ The 5DE is constructed from individual level empowerment scores which reflects each person's achievements in the 5 sub‐outcomes (see Table 2) and measured by the 10 indicators with their corresponding weights. Each indicator measures whether an individual has surpassed a given threshold, or has adequate achievement, with respect to each indicator.

^f^ As noted by Malapit and Quisumbing (2015), gender parity is not equal to gender equality.

^g^ Gender Equality: Gender equality refers to the state in which individuals, regardless of gender, have equal rights, responsibilities, and opportunities across all aspects of life. It seeks to eliminate discrimination based on gender and address systemic inequalities, ensuring that everyone can participate fully in society, including access to education, health, and employment (UNDP KYRGYZSTAN 2023). Gender equality is critical for sustainable development, enhancing the well‐being and prosperity of communities worldwide (OECD 2023).

^h^ Gender Assets Gap Project (Cf. Deere et al. 2013).

^i^ According to Vereinte Nationen (2015), unsafe market spaces, transport, and public spaces expose women workers and traders to violence, and limit their economic opportunities.

The EGM did not code adverse and unintended outcomes separately, but included all the studies that also reported on adverse or unintended outcomes on nutritious diets and gender outcomes. The sub‐domains of the outcomes were labeled and defined as neutral and bidirectional to accommodate both the intended and unintended (adverse) outcomes.

### 3.10 Other Eligibility Criteria

**Figure 2:**
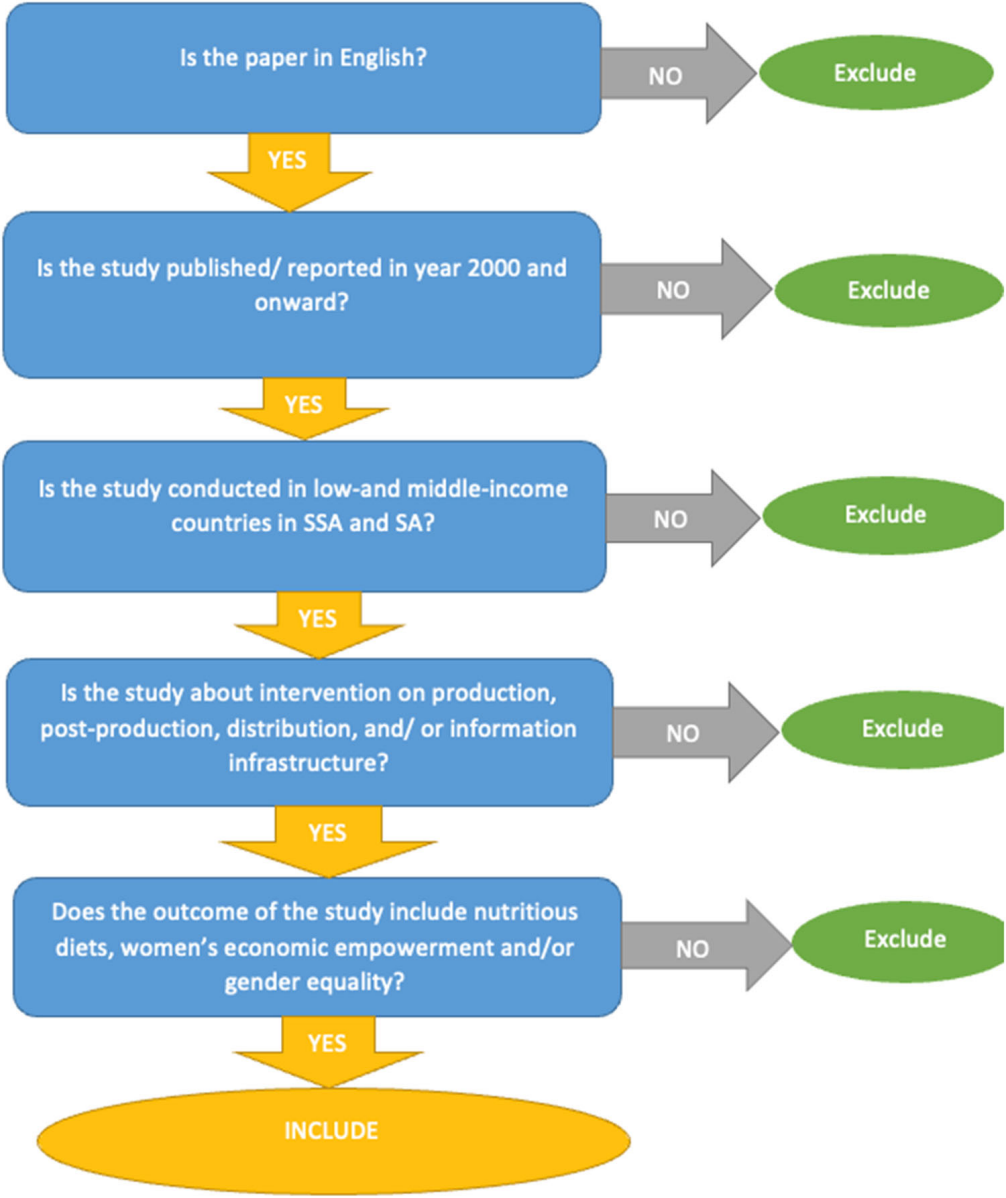
Screening tool for the EGM.

There were two other eligibility criteria for including studies in the EGM: time frame and language. We included only studies that had been published (reported) in Year 2000 and onwards and written in English languages. This means that studies that were published before 2000 and written in languages other than English were excluded from the EGM (see Figure 2 for details of the screening/exclusion criteria).

The chosen timeframe of 2000 (including ongoing studies) reflects significant regional developments related to infrastructure, nutrition, and gender equality. Starting from the Year 2000 aligns with the launch of the Millennium Development Goals, a period marked by increasing global attention and investment in sustainable development, infrastructure, and gender issues in LMICs. Extending the timeframe to include ongoing studies ensures that the EGM captures the most up‐to‐date and relevant evidence, including emerging data that might not yet be formally published but contributes to the growing body of knowledge.

### 3.11 Search Methods and Sources

The Protocol used to guide the development of this EGM was published in the Campbell Systematic Reviews database (Odei Obeng‐Amoako et al. 2023).

#### 3.11.1 Search Strategy

We adapted a standardized search strategy that identified published and unpublished studies for this EGM in 3 academic databases, 33 institutional websites, Google, Google Scholar, and 8 registries of randomized control trials and pre‐analysis plans (Appendices A and B). Through Machine Learning in OpenAlex in EPPI‐Reviewer software, additional studies were also identified for this EGM. The machine learning process is such that it automatically analyzed and categorized the millions of research articles to identify relevant studies. This included training algorithms on keywords and topics related to our EGM, which helped in filtering and retrieving studies that met our search criteria, thereby making evidence gathering efficient and comprehensive. The search strategy and search terms were pre‐tested repeatedly to ensure the certainty of its ability to identify all potential published and unpublished papers for the EGM. The search terms were developed based on population, interventions, and outcomes (PIO) (see Appendix C for search terms). This allowed easy and effective retrieval of potential studies from the databases for the EGM. Search terms were not restricted by study designs because this EGM covered all types of study designs that reported empirical evidence as well as review of literature. However, study designs were used as filters in displaying the EGM.

Peer‐reviewed articles were searched from scientific and academic databases: the CAB Abstract, GreenFILE EBSCO, and Medline PubMed (refer to Appendix D for search terms and results). Searches in the institutional websites, Google, Google Scholar, and registries identified both published and unpublished for this EGM. The rationale for limiting searches in these few databases was because we conducted additional searches for more studies using Machine Learning in OpenAlex in EPPI‐Reviewer software (Thomas et al. 2010). OpenAlex is an index of hundreds of millions of interconnected entities across the global research system (Priem et al. 2022). The search for studies in OpenAlex was conducted after the full‐text screening of identified studies in academic and organizational databases searches. This software helped to effectively use resources, including time, in developing the EGM.

#### 3.11.2 Other Search Strategies

Additional searches for eligible studies were carried alongside the screening and coding of studies earlier identified for the EGM. Subsequently, we searched for additional studies through hand search, backward citation of identified systematic reviews, and contacting key authors of relevant papers identified for the EGM. We hand‐searched the ANH Academy for proceedings of conferences and abstracts databases, evaluation reports, and academic theses, existing relevant EGMs conducted by organizations such as Campbell Collaboration and 3ie for eligible studies for EGM (see Moore et al. 2021; Malhotra et al. 2021; Sparling et al. 2022). Five authors with relevant papers were contacted to inquire about their additional studies or other studies they were aware that could be relevant to the present EGM. Three of the five authors responded to our enquiries and shared some papers for the EGM (See Appendix D). A log of literature search activities was kept for reporting purposes. All the eligible papers were converted into Research Information System (RIS) and uploaded into EPPI‐Reviewer software (Thomas et al. 2010).

#### 3.11.3 Protocol Modification and Supplementary Search

Following the submission of the standardized protocol to the Campbell Systematic Review Journal, minor modifications were made to the search strategy. The modifications included: (i) removal of green infrastructure from the type of infrastructure and (ii) addition of greenhouse and Agrivoltaic as types of infrastructure. We rescreened and excluded studies that were earlier included for green infrastructure. We also conducted a supplementary search for studies on greenhouse and Agrivoltaic in Google and Google Scholar databases, and also searched for more studies through machine learning in OpenAlex database in the EPPI‐Reviewer software. We also conducted a title and abstract, and full‐text screening of previously excluded studies to make sure that studies from the original search with a focus on greenhouse and Agrivoltaic were included.

### 3.12 Data Collection and Analysis

#### 3.12.1 Screening and Study Selection

All references of identified papers were uploaded into EPPI‐Reviewer software and were de‐duplicated. Screening of eligible papers was conducted in two phases. First, we screened the title and abstract of the papers to assess their eligibility for the EGM. To make the title and abstract screening faster, we adopted a machine learning model to order the identified papers by relevance or priority based on the defined eligibility criteria for the EGM (refer to Figure 2). We conducted a single screening for the title and abstract for the identified papers after the team had undergone a series of standardization exercises or training until there was an 85% agreement on the eligibility (i.e., inclusion and exclusion criteria) of a set of identified papers by the reviewers.

Second, we conducted a full‐text screening of all the papers included in the title and abstract screening that were retrieved and screened by two reviewers independently (double screening). Reports on the full‐text screening were generated for comparison among the reviewers. The team of reviewers discussed and reconciled the reports in EPPI‐Reviewer. In case of any misunderstanding regarding the eligibility of a paper, a third reviewer assisted to resolve the differences. Subsequently, the eligible papers for the EGM at the full‐text screening were coded based on a pre‐defined data extraction form in EPPI‐Reviewer by two independent reviewers (see Appendix E for coding form). The pre‐defined data extraction was accompanied by the definitions of codes and examples of interventions, outcomes, types of evaluations, study designs, and list of LMIC countries per the World Bank classification. The reports of coded data were compared and reconciled by the reviewers in EPPI‐Reviewer.

#### 3.12.2 Data Management and Analysis

The coded data set in EPPI‐Reviewer was cleaned for data analysis and the generation of the EGM. Outliers of codes and missing codes were identified by univariate and bivariate analysis in EPPI‐Reviewer. The reasons for the deviations were identified and rectified by the team. A random sample of coded data and papers was validated repeatedly by an in‐house team. All missing papers were identified, and the corresponding authors were contacted for full reports. Analysis of descriptive statistics was conducted in Microsoft Excel and STATA version 16.

### 3.13 Analysis and Presentation

#### 3.13.1 Report Structure

The current report is structured into eight sections: the abstract, plain language summary, background, objectives, methods, results, discussion, and conclusion. Each section also has appropriate sub‐sections. The report contains 13 tables and 12 figures. The report also contains eight appendices (Table 3).

**Table 3:** Report structure.

**Figure 3:**
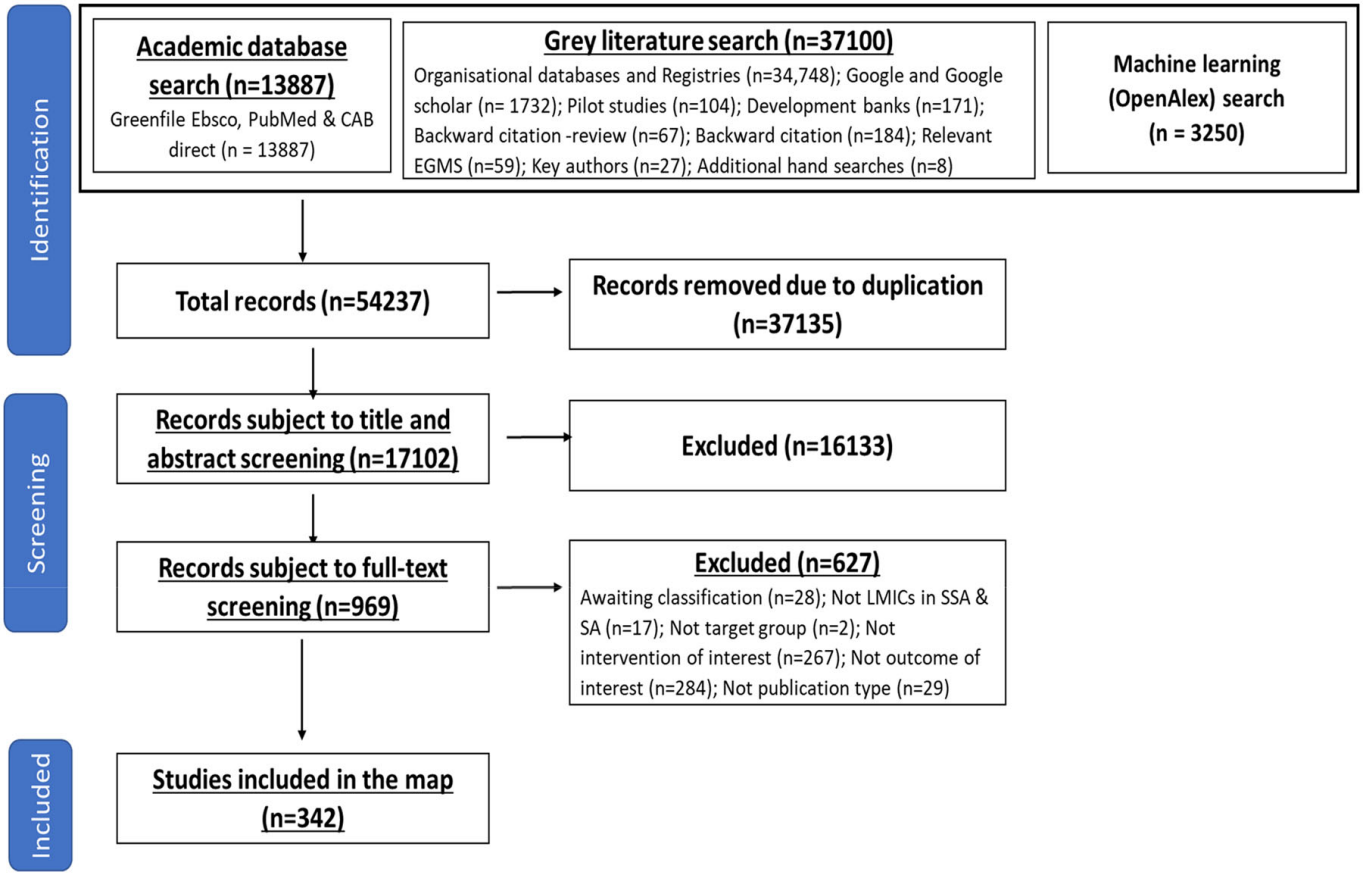
PRISMA flowchart.

**Figure 4:**
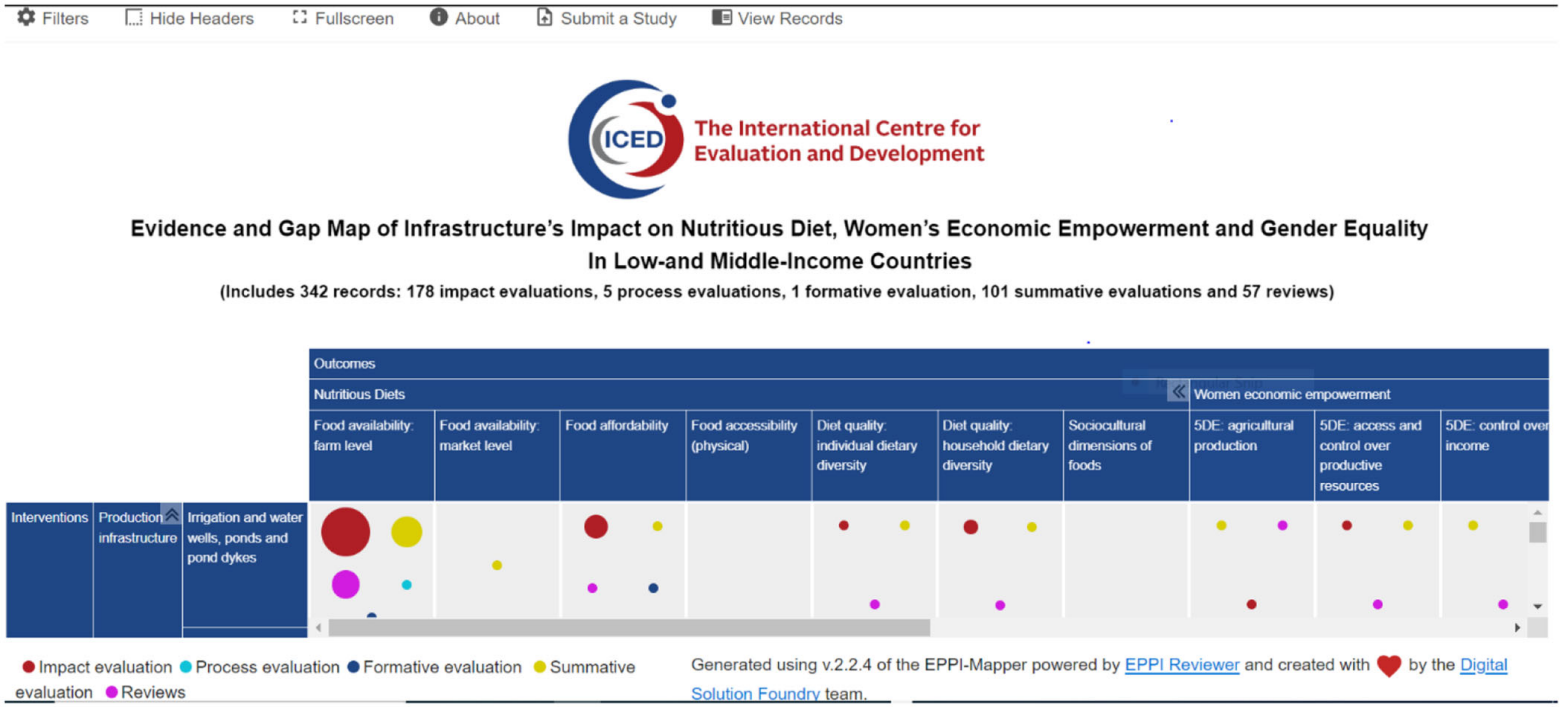
A snapshot of EGM. The EGM can be found on https://products.iced-eval.org/egm-iindwege.html.

**Figure 5:**
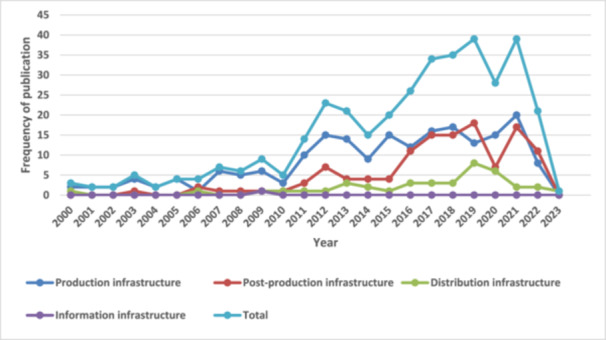
Trends of publications by interventions from Years 2000 to 2023.

Abstract
Abbreviations and acronyms
Plain language summary
Background
Objectives
Methods
Figure 1: Evidence Based Theory of Change
Table 1: Categories, sub‐categories, and intervention examples (infrastructure)
Table 2: Sub‐domains of outcomes with examples
Figure 2: Screening tool for the EGM
Table 3: Report structure
Table 4: Filter dimensions and their descriptions
Results
Figure 3: PRISMA flowchart
Table 5: Reasons for exclusion of studies at full text screening
Table 6: Examples of excluded studies
Figure 4: A snapshot of EGM.
Figure 5: Trends of publications by interventions from Years 2000 to 2023
Figure 6: Trends of publications by outcomes from Years 2000 to 2023
Table 7: Frequency of types of publications of studies included in the EGM
Table 8: Frequency of type of evaluation in included studies
Figure 7: Frequency of types of study designs in the included studies
Figure 8: Frequency of geographic regions in the included studies
Figure 9: Geographic distribution of included studies
Figure 10: Frequency of intervention categories cited in the included studies (*n* = 370)
Table 9: Frequency of categories and sub‐categories of interventions cited in the included studies
Figure 11: Frequency of outcome categories cited in the included studies
Table 10: Frequency of categories and sub‐categories of outcomes in the included studies
Table 11: Aggregate map of intervention by outcome categories of the included studies
Table 12: Distribution of outcome and intervention categories versus types of study designs among the included studies
Figure 12: Distribution of evidence on outcome and intervention categories by types of evaluation among the included studies
Table 13: Distribution of evidence on intervention and outcome categories versus geographical regions
Discussion
Potential biases in the mapping process
Authors conclusion
Acknowledgment
References

#### 3.13.2 Filters for Presentation

The EGM uses several dimensions as a filter so that users of the map can restrict and display the map for only filtered studies. The dimensions include country and region of study, study design, type of evaluation, year of publications, type of publication, and study status (see Table 4 for the description of the filter variables).

#### 3.13.3 Dependency/Unit of Analysis

The unit of analysis was a study, meaning that each item presented in the EGM represented a study. A study was defined as an analysis of a unique sample, which included multiple time points for the same sample (White et al. 2018). Like earlier EGMs, if a single study had multiple publications, we chose the most recent open‐access publication for the EGM (Malhotra et al. 2021). If the study reported on multiple interventions, outcomes, or study designs, it was coded multiple times per the appropriate coding category. Therefore, studies with multiple codes for interventions, outcomes, or study designs were counted as one entry in the EGM. Furthermore, if a study had multiple interventions or outcomes, with some being ineligible for the EGM, the appropriate interventions or outcomes qualified for the EGM were coded. It is worth to note that as a result, multiple coding of multiple interventions, outcomes, or study designs, frequencies of codes may not match the total number of eligible studies in EGM.

**Table 4:** Filter dimensions and their descriptions.

Dimensions of filter	Description
Region	List of study regions according to the World Bank categorization. We have five regions: South Asia, Eastern, Western, Southern, and Central Africa.
Country	List of countries in the study regions are presented in the EGM. We have a total of 54 countries: 8, 10, 20, 9, and 7 South Asia, Eastern, Western, Southern, and Central Africa, respectively.
Types of evaluation	The types of evaluation used as filters in the EGM include impact, process, summative, formative evaluations and reviews.
Study design	Types of study designs employed by the studies were set as filters. The EGM considered four study designs: experimental, non‐experimental (including quasi experimental, regression‐based studies), qualitative, and systematic reviews, scoping reviews and other reviews.
Year of publication	The EGM also reports the date paper was published in a journal and not when it was indexed online. The current EGM included year of publications ranging from 2000 to 2023. However, few papers were included in the EGM that had no date of publication.
Type of publication	This includes the list of the publication outlet of the studies. We considered eight types of publications: peer‐reviewed article, preprint in peer‐reviewed journal, report, working paper, conference paper, discussion paper, dissertation, and protocol.
Status of studies	The publication status of the studies, and we considered two categories of study completion status: completed and ongoing.

The inclusion of a systematic review was assessed based on our eligibility criteria. The individual primary studies included in the systematic review were assessed to inform the decision on inclusion. However, primary studies appearing in a systematic review that qualified for our EGM were considered as a unit of analysis and counted once. Following, Apunyo et al. (2022), our EGM classified the clusters of evidence as highly dense or well‐evidence/research (*n* ≥ 75), moderately dense or evidence/researched (*n* = 75 – 25) and less dense or less evidence/researched (< 25) for the frequencies of the characteristics of the codes.

### 3.14 Data Collection and Methods

#### 3.14.1 Methods for Mapping

All the coded data for the eligible studies were exported from EPPI‐Reviewer in JavaScript Object Notation (Json) data format. The data was then imported into EPPI‐Mapper, where the EGM was produced.

## 4 Results

### 4.1 Results of the Search

A total of 54,237 studies were identified from academic databases, gray literature, and machine learning sources, including OpenAlex in EPPI‐Reviewer. After removing 37,135 duplicates, 17,102 studies underwent title and abstract screening, resulting in 969 studies retained for full‐text review. Out of these, 342 studies met eligibility criteria and were coded for inclusion in the evidence map (see Figure 3).

### 4.2 Excluded Studies

#### 4.2.1 Reason for Excluding Studies

Studies were excluded at the full‐text screening for various reasons as shown in Table 5. Some of the reasons for excluding papers were ineligible study population (*n* = 17), ineligible intervention (*n* = 267), and ineligible outcome (*n* = 284).

**Table 5:** Reasons for exclusion of studies at full‐text screening.

Studies	Frequency (*n*)	Share (%)
Included	342	35.3
Studies conducted in LMICs in SSA and SA	19	2.0
Studies about interventions on production, post‐production, distribution and/or information infrastructure	267	27.6
Outcome of the study include nutritious diet, women economic empowerment and gender equality.	287	29.3
Publication type	29	3.0
Awaiting classification	28	2.9
Total	969	100

#### 4.2.2 Studies Awaiting Classification

Table 6 shows the characteristics of studies identified as potentially eligible but have not been incorporated into the map with various reasons for exclusion.

**Table 6:** Examples of excluded studies.

Author	Title	Excluded on
Michelle Nayahamui Rooney (2016)	Women's economic empowerment: the importance of small	Country (SSA and SA)
	market stall vendors in urban Papua New Guinea	
Matita et al. (2021)	Does household participation in food markets increase dietary diversity?	Intervention
	Evidence from rural Malawi	
Schreinemachers et al. (2019)	Impact of school gardens and complementary nutrition education in Burkina Faso	Intervention
Aku et al. (2018)	Effect of market access provided by farmer organizations on smallholder vegetable farmer's income in Tanzania	Outcome
Bensch et al. (2011)	Impacts of Rural Electrification in Rwanda	Outcome

### 4.3 Synthesis of Included Studies

#### 4.3.1 Characteristics of the EGM

**Figure 6:**
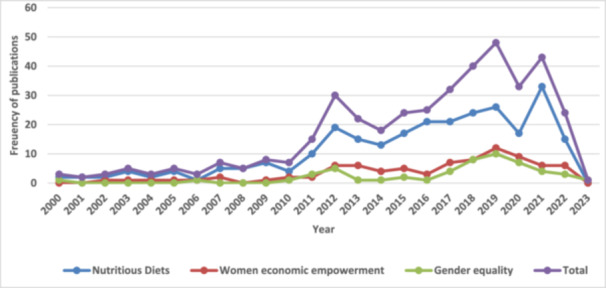
Trends of publications by outcomes from Years 2000 to 2023.

The current interactive EGM comprises 342 studies, including 337 completed studies and 5 ongoing studies from LMICs across SSA and SA (Figure 4). This map provides an overview of existing evidence and identifies gaps concerning the impact of infrastructure on nutritious diets, women's economic empowerment, and gender equality from 2000 to 2023. Results indicate a notable increase in publications related to infrastructure beginning in 2010 (Figure 5). A gradual increase in publications on women's economic empowerment and gender equality is also observed from 2010, peaking in 2019 (Figure 6). Figures 5 and 6 highlight completed and published studies from 2000 onward, excluding ongoing studies without publication dates. The majority of studies included in the evidence gap map are peer‐reviewed articles (72.2%), with reports comprising 12.9% and academic dissertations 5.8% (Table 7).

**Table 7:** Frequency of types of publications of studies included in the EGM (*n* = 342).

Types of publications	Frequency (*n*)	Percent (%)
Peer‐review article	247	72.2
Report	44	12.9
Working paper	14	4.1
Conference paper	3	0.9
Discussion paper	9	2.6
Dissertation	20	5.8
Protocol	5	1.5
Total	342	100

#### 4.3.2 Type of Evidence

**Figure 7:**
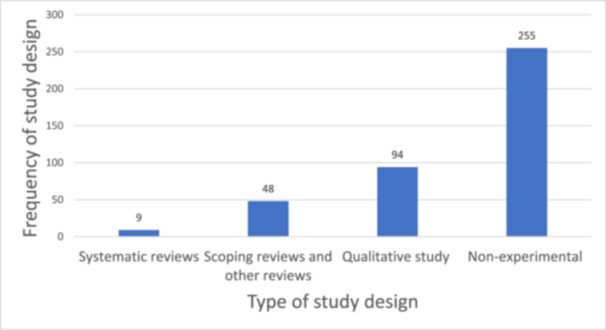
Frequency of types of study designs in the included studies (*n* = 406). Non‐experimental study designs include quasi‐experimental, descriptive, and regression‐based studies.

The majority of the studies included in the EGM were impact evaluations (*n* = 178; 52.0%) and summative evaluations (*n* = 101; 29.5%). Few of the included studies were process evaluations (*n* = 5) and formative evaluations (*n* = 1) (Table 8). In terms of study designs, most of the included studies were non‐experimental studies (*n* = 255), followed by qualitative studies (*n* = 94) (Figure 7). Few of the included studies applied systematic reviews methods without meta‐analysis (*n* = 9) to generate evidence on impact of infrastructure on nutritious diet, women's economic empowerment, and gender equality (Figure 7). There were no experimental studies in the EGM.

**Table 8:** Frequency of type of evaluation in included studies.

Type of evaluation	Frequency (*n*)	Percent (%)
Impact evaluation	178	52.0
Process evaluation	5	1.5
Formative evaluation	1	0.3
Summative evaluation	101	29.5
Reviews	57	16.7
Total	342	100.0

#### 4.3.3 Evidence by Population

**Figure 8:**
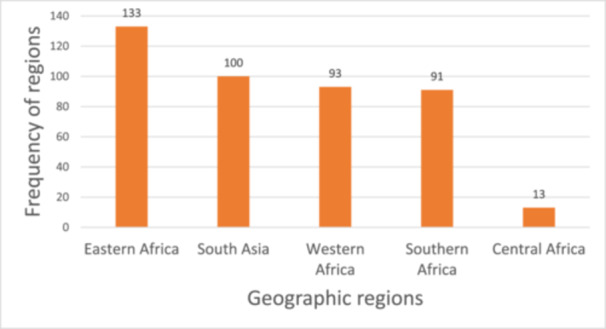
Frequency of geographic regions in the included studies (*n* = 430).

The Eastern Africa region (*n* = 133) had the highest concentration of evidence on the relationship between infrastructure, nutritious diets, women's economic empowerment, and gender equality, compared with other geographic regions (Figure 8). The SA region (*n* = 93) had slightly less evidence than the Western (*n* = 100) and Southern Africa (*n* = 91) regions. The Central Africa region had the least representation (*n* = 13) among the studies included in the EGM (Figure 8).

**Figure 9:**
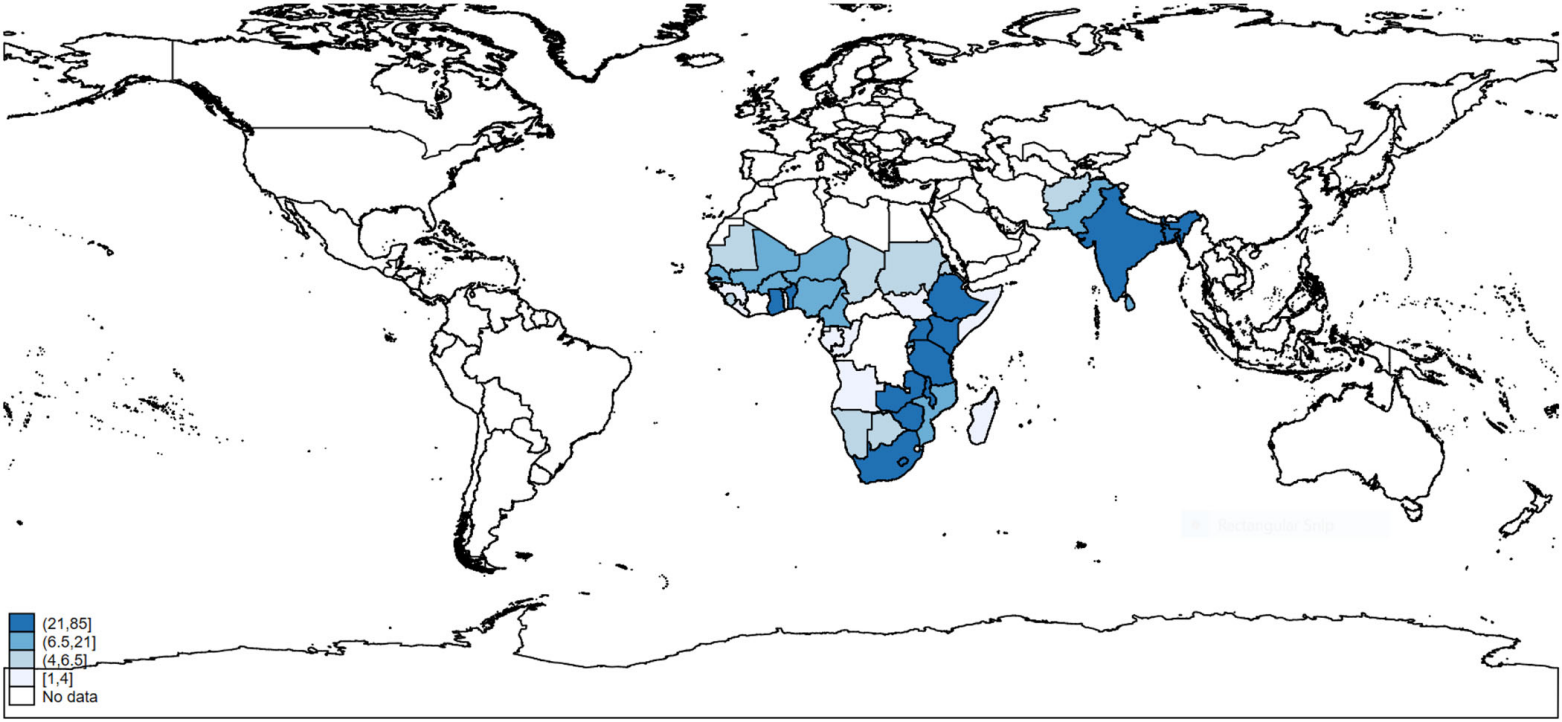
Geographic distribution of included studies.

Regarding the distribution of evidence by country, all 54 countries involved in the EGM had at least one study recorded. Ethiopia was the most studied country (*n* = 85), followed by India (*n* = 54), Ghana (*n* = 46), Kenya (*n* = 44), South Africa (*n* = 36), Bangladesh (*n* = 32), and Nepal (*n* = 27). Conversely, Comoros (*n* = 1), Cape Verde (*n* = 1), Sao Tome and Principe (*n* = 1), and the Maldives (*n* = 1) were the least frequently studied populations in this EGM (Figure 8; Supporting Information S1: Result 1). Figure 9 presents a heat map indicating the geographical distribution of the included studies.

#### 4.3.4 Intervention Categories

**Figure 10:**
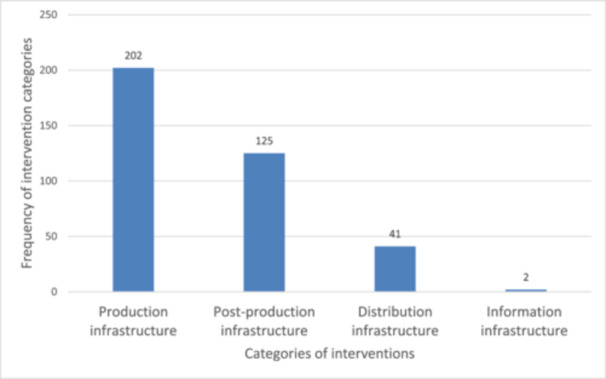
Frequency of intervention categories cited in the included studies (*n* = 370).

Four infrastructural intervention categories were involved in this EGM (Figure 10). Production infrastructure (*n* = 202) was the most researched category among the included studies. Few studies focused on information infrastructure (Figure 10). The results show that irrigation and water wells, ponds and pond dykes (*n* = 226) were the most researched sub‐categories in the production category (Table 8). Market facilities (*n* = 70) in the post‐production category had the highest frequency of being studied among the included studies. Road infrastructure (*n* = 37) was most researched in the distribution infrastructure category in the included studies (Table 9). The least studied intervention was information infrastructure in the included studies (Figure 10 and Table 9). Some of the evidence gaps included limited studies on greenhouse (*n* = 2), on‐farm energy and power supply (*n* = 5) in the production category, processing facilities (*n* = 2) and storage facilities (*n* = 11) in the post‐production category and railways (*n* = 1) and bridges (*n* = 5) in the distribution infrastructure category (Table 9).

**Table 9:** Frequency of categories and sub‐categories of interventions cited in the included studies.

Categories and sub‐categories of interventions	Frequency (*n*)
Production (*n*)	202
Irrigation and water wells, ponds and pond dykes	202
Greenhouse	2
On‐farm energy and power supply (solar, wind, water, agrivoltaic)	5
Post‐production infrastructure (*n*)	125
Market facilities	70
Processing facilities (grain mills, solar dryer)	2
Storage facilities (Cold room and warehouses)	11
Off‐farm energy and power supply (solar, wind, water)	47
Distribution infrastructure (*n*)	41
Roads	37
Railways	1
Bridges	5
Information infrastructure (*n*)	2
Information centers: community radios stations and information boards	1
Telecommunication masts	2

#### 4.3.5 Outcome Categories

**Figure 11:**
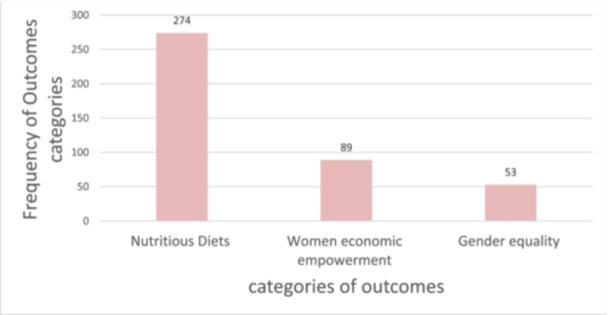
Frequency of outcome categories cited in the included studies (*n* = 416).

The outcomes of interest for the EGM were in three categories: nutritious diet, women's economic empowerment, and gender equality (Figure 11). Nutritious diet outcome (*n* = 274) was the most studied, compared with women's economic empowerment (*n* = 89) and gender equality outcomes (*n* = 53) included in the EGM. In terms of evidence by sub‐categories of outcomes, diet quality (individual dietary diversity) (*n* = 30) and diet quality (household dietary diversity) (*n* = 70) were least frequently studied outcomes compared with food availability at the farm level (*n* = 189) and food affordability (*n* = 81) in the nutritious diet outcome category. Income and employment (*n* = 50) and economic opportunities and outcomes (*n* = 34) were predominantly researched in the women's economic empowerment and gender equality outcome categories, respectively. Limited evidence exists on food availability at the market level (*n* = 6), gender parity index (GPI) (*n* = 4) and exposure to risk (*n* = 6) in the nutritious diets, women's economic empowerment, and gender equality categories, respectively (Table 10).

**Table 10:** Frequency of categories and sub‐categories of outcomes in the included studies.

Outcome category and sub‐categories	Frequency (*n*)
Nutritious diet outcome	274
*Sub‐categories*	
Food availability: farm level	189
Food availability: market level	6
Food affordability	81
Diet quality: individual dietary diversity	28
Diet quality: household dietary diversity	70
Social dimensions of food	0
Women economic empowerment outcome	89
*Sub‐categories*	
5DE: agricultural production	20
5DE: access and control over productive resources	39
5DE: control over income	27
5DE: time	39
5DE: leadership	17
WEAI (pro‐WEAI, A‐WEAI)	8
Gender parity index (GPI)	4
Income and employment	50
Gender equality outcome	53
*Sub‐categories*	
Economic opportunities and outcomes	34
Social outcomes	25
Leadership, agency, and collective action	12
Exposure to risk	6

#### 4.3.6 Aggregate Map of Evidence: Outcome Versus Intervention Categories

Table 11 presents an aggregate map that organizes interventions by outcome categories, showing the number of studies included in each combination. It lists four primary intervention categories: Production Infrastructure, Post‐Production Infrastructure, Distribution Infrastructure, and Information Infrastructure, each aimed at addressing various outcome categories. The outcome categories are: Nutritious Diet, Women's Economic Empowerment, and Gender Equality, each with a corresponding number of studies focused on them. For Production Infrastructure, there are 188 studies related to Nutritious Diet, 36 on Women's Economic Empowerment, and 13 on Gender Equality. Post‐Production Infrastructure includes interventions aimed at improving storage, processing, and other post‐harvest activities, with 87 studies for Nutritious Diet, 45 for Women's Economic Empowerment, and 25 for Gender Equality. In the Distribution Infrastructure, there are 23 studies related to Nutritious Diet, 13 to Women's Economic Empowerment, and 17 to Gender Equality. Finally, Information Infrastructure, only 2 studies related to Nutritious Diet, 1 for Women's Economic Empowerment, and none for Gender Equality.

**Table 11:** Aggregate map of intervention by outcome categories of the included studies.

Outcome categories			
Intervention categories	Nutritious Diet (*n*)	Women's economic empowerment (*n*)	Gender equality (*n*)
Production infrastructure	188	36	13
Post‐production infrastructure	87	45	25
Distribution infrastructure	23	13	17
Information infrastructure	2	1	0

#### 4.3.7 Distribution of Evidence on Outcome and Intervention Categories by Study Designs Among the Included Studies

**Figure 12:**
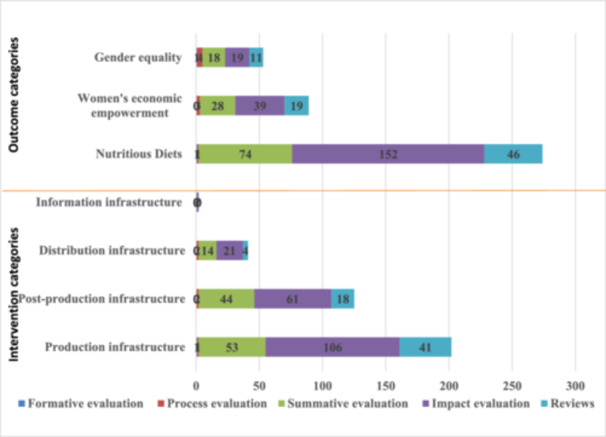
Distribution of evidence on outcome and intervention categories by types of evaluation among the included studies.

Majority of the included studies used non‐experimental study designs to assess the effect of infrastructural intervention on the various outcomes of interest. The frequency of qualitative study design used was 135 for outcome and 99 for intervention categories in the included studies. Few systematic reviews were conducted on outcome (*n* = 12) and intervention (*n* = 12) categories (Table 12). None of the studies included in the EGM used an experimental study design or randomized control trial. Majority of included studies reporting on nutritious outcome category were impact evaluation (*n* = 152), followed by summative evaluation (*n* = 74) (Figure 12). Similarly, most of the studies that reported on production infrastructure were impact evaluations (*n* = 106). Generally, process evaluation and formative evaluation were least reported across the various interventions and outcome categories among the included studies in the EGM (Figure 12).

**Table 12:** Distribution of outcome and intervention categories versus types of study designs among the included studies.

	Study designs				
Attributes	Experimental (*n*)	Non‐experimental (*n*)	Qualitative (*n*)	Systematic reviews(*n*)	Scoping reviews and other reviews (*n*)
Outcome categories					
Nutritious diets	0	215 (72.9)	60 (44.4)	8 (66.7)	38 (59.4)
Women's economic empowerment	0	49 (16.6)	46 (34.1)	3 (25.0)	16 (25.0)
Gender equality	0	31 (10.5)	29 (21.5)	1 (8.3)	10 (15.6)
Total	0	295 (100.0)	135 (100.0)	12 (100.0)	64 (100.0)
Intervention categories					
Production infrastructure	0	148 (53.8)	49 (49.5)	6 (50.0)	35 (68.6)
Post‐production infrastructure	0	92 (33.5)	36 (36.4)	5 (41.7)	13 (25.5)
Distribution infrastructure	0	33 (12.0)	13 (13.1)	1 (8.3)	3 (5.9)
Information infrastructure	0	2 (0.7)	1 (1.0)	0 (0.0)	0 (0.0)
Total	0	275	99 (100.0)	12 (100.0)	51 (100.0)

#### 4.3.8 Distribution of Evidence on Intervention and Outcome Categories by Geographical Regions

Table 13 shows the distribution of intervention and outcome categories by geographic regions among the included studies of the EGM. The Eastern Africa region had most studies for both interventions (*n* = 143) and outcomes (*n* = 157), while the Central Africa region had the least records of studies for both interventions (*n* = 13) and outcomes (*n* = 15) in the EGM (Table 13). Ethiopia was most studied on production infrastructure (*n* = 58) and nutritious diets (*n* = 80) (Supporting Information S3: Result 3 and Supporting Information S4: Result 4). Detailed analysis of the results showed that majority of the studies in Ethiopia, Ghana and India were on irrigation and water wells, ponds and pond dykes. Studies on information infrastructure were found in Uganda (*n* = 1) and Ghana (*n* = 1) (Supporting Information S5: Result 5). Ethiopia (*n* = 24) also had the highest record of studies on market facilities, followed by Kenya (*n* = 13) and Malawi (*n* = 12) among the included studies in the EGM (Supporting Information S5: Result 5). Food availability at the farm level was the study outcome in studies conducted in Ethiopia (*n* = 49), Kenya (*n* = 25), and India (*n* = 35). Ethiopia had the most studies reporting on household dietary diversity (*n* = 23) and individual dietary diversity (*n* = 13) (Supporting Information S6: Result 6).

**Table 13:** Distribution of evidence on intervention and outcome categories versus geographical regions.

Attributes	Geographical regions				
	Central Africa (*n*, %)	Eastern Africa (*n*, %)	Western Africa (*n*, %)	Southern Africa (*n*, %)	South Asia (*n*, %)
Intervention categories					
Production infrastructure	8 (61.5)	77 (53.8)	66 (62.9)	58 (60.4)	51 (45.1)
Post‐production infrastructure	4 (30.8)	54 (37.8)	27 (25.7)	31 (32.3)	39 (34.5)
Distribution infrastructure	1 (7.7)	11 (7.7)	11 (10.5)	7 (7.3)	23 (20.4)
Information infrastructure	0 (0.0)	1 (0.7)	1 (1.0)	0 (0.0)	0 (0.0)
Total	13 (100.0)	143 (100.0)	105 (100.0)	96 (100.0)	113 (100.0)
Outcome categories					
Nutritious diet	13 (86.7)	118 (75.2)	78 (64.5)	72 (64.3)	68 (52.7)
Women's economic empowerment	1 (6.7)	27 (17.2)	29 (24.0)	24 (21.4)	35 (27.1)
Gender equality	1 (6.7)	12 (7.6)	14 (11.6)	16 (14.3)	26 (20.2)
Total	15 (100.0)	157 (100.0)	121 (100.0)	112 (100.0)	129 (100.0)

### 4.4 Discussion

#### 4.4.1 Summary of Main Results

##### 4.4.1.1 Main Findings

The present EGM shows available evidence and gap on the linkages between physical infrastructure and nutritious diets, women's economic empowerment and gender equality in LMICs in SSA and SA regions. The EGM shows a steady growth in evidence over the last two decades. We identified 342 studies, including 5 ongoing studies from 54 countries across SSA and SA regions. Eastern African region had the highest number of literature on the relationship between physical infrastructure and nutritious diets, women's economic empowerment and gender equality compared with Southern, Western, Central Africa and SA regions. Production infrastructure category was the most researched among the different infrastructure categories, while nutritious diets were the most studied among the outcome categories. Generally, evidence on how physical infrastructure relates to women's economic empowerment and gender equality was sparse. The linkage between production infrastructure and nutritious diets outcome had the most clusters of evidence and a potential area for future evidence synthesis. Studies identified in the EGM were mostly impact evaluations and summative evaluations. Few process and formative evaluations were found in the EGM.

#### 4.4.2 Areas of Clusters of Evidence and Major Gaps in the Evidence

*Identify and describe clusters of evidence gaps (ER10). Use EGM framework.*

Eastern African Region was the most frequent studied region in the EGM probably because Ethiopia, Kenya and Tanzania were often cited as study locations in studies about the effects of irrigation and water wells, ponds and pond dykes on farm‐level food availability. Similarly, India and Bangladesh were often mentioned as study locations in studies conducted on irrigation and water wells, ponds and pond dykes and farm‐level food availability in the South Asian region. These findings are very useful to inform climatic change adaptation for sustainable agriculture and food systems policy themes because reports show that irrigation infrastructure makes agricultural production possible year‐round even in desert, arid, and semi‐arid settings in Africa and Asia (Gunston 2007). For instance, in Namibia, Kenya, and Sudan, irrigation systems stimulate rainfed conditions in desert, semi‐desert and low‐productivity rangeland to produce fruits and cotton. In South Africa and other SSA countries, irrigation has been utilized to raise the productivity of existing crop production, most notably the production of maize and vegetables (Gunston 2007). This finding justifies the need for systematic reviews on how irrigation impacts food availability to inform irrigation interventions, especially in arid and semi‐arid settings with poor rainfall patterns and poor food security in SSA and SA regions.

Notably, evidence on production infrastructure was saturated around irrigation and water wells, ponds, and pond dykes and food availability at the farm level. However, evidence on irrigation and water wells, ponds and pond dykes on food affordability, household and individual dietary intake was moderately dense. Clearly, evidence gaps exist on effects of irrigation on market food availability and individual dietary intake. Literature shows irrigation infrastructure increases food availability and potentially reduces under‐nutrition among rural households (Hopea et al. 2008; Pandey et al. 2016). Given the growing evidence shown in this EGM, future systematic reviews should consider the effects of irrigation and nutritious diets.

Investment in irrigation infrastructure could contribute to improve women's economic empowerment. Shah et al. (2014) show that irrigation infrastructure enables landless and poor women to engage in agricultural wage labor. Furthermore, irrigation infrastructure could improve time‐use efficiency, stabilize and increase income, and boost women's status in the household and communities in LMICs (Theis et al. 2016). However, this EGM shows little evidence on the irrigation infrastructure and the various WEAI dimensions. For instance, few studies reported on irrigation infrastructure and agricultural production, access and control over productive resources, control over income, time use, and leadership. This finding is an alert to research commissioners to focus their research work on production infrastructure and the WEAI dimensions.

Post‐production infrastructure, including market facilities, storage, and food processing facilities, could contribute to improve the ability of food systems to make nutritious diets affordable, accessible, and safe for LICs and subsequently empower women. The moderate evidence on the influence of market facilities on food availability, food affordability, and dietary intake shows an emerging research interest in the field of sustainable food systems. Probably, having access to markets provides access to food year‐round and subsequently improves diverse dietary intake. Therefore, future evidence synthesis to consolidate results of existing studies on markets and nutritious diets will be useful for future policy‐making decisions. It is worth noting that few studies have focused on post‐production infrastructure and women's economic empowerment pathways as reported in this EGM. This was surprising considering the enormous extant evidence on the roles women play in agricultural and food systems (Malapit and Quisumbing 2015; Mohun and Biswas 2016). Furthermore, women's participation in economic activities such as marketplace enhances their wellbeing and economic development, and contributes to the development of their communities and nation as a whole. A report by UN Women shows about 75%–90% of all market vendors in the Pacific are women; despite the long hours and poor working conditions, their earnings contribute significantly to the incomes of their households (UN Women Asia Pacific 2018). Therefore, given the evidence gap, future primary studies on the effects of market facilities, storage, and food processing facilities on women's economic empowerment and gender equality would prove useful for decision‐making.

It was observed that most of the studies in the post‐production infrastructure category focused on the effects of off‐farm energy and power supply mainly generated by solar, wind, water (hydro‐electric power) on women's use of time, access and control over productive resources and control over income under the women's economic empowerment outcome category. Evidence on women's use of time, income, and employment, and the linkage with energy and power supply intervention, was common in the literature. Reports show that about 35% and 73% of the populations in SSA and SA regions have access to electricity, respectively (Castalia Strategic Advisors 2014). Therefore, evidence synthesis on the relationship between off‐farm energy and power supply and women's economic empowerment will provide useful information needed to incentivize governments and other donors to prioritize investments in off‐farm energy and power supply infrastructure.

Moderate evidence on distribution infrastructure (roads, railways, and bridges) exists in the literature. A handful of studies focused on the association between roads and food availability at the farm level and household dietary diversity. On the other hand, studies on roads reported on income and employment, equal economic opportunities for men and women, and social outcomes. Extant reports show a deficit in road access coverage, and only 31% of rural populations in SSA region and 58% of rural population in SA region have access to all‐weather roads, respectively (Castalia Strategic Advisors 2014). Therefore, evidence gaps on distribution infrastructure identified in this EGM have implications for the need to commission more studies on the impact of roads, railway, and bridges on nutritious diets, women's economic empowerment, and gender equality. Findings of future studies will inform decision‐making on investments on distribution infrastructure.

Generally, evidence on information infrastructure was scanty, probably the emphasis on physical presence of telecommunication mast, information center, community information boards, and community radio stations, and their effects on nutritious diet, women's economic empowerment, and gender equality as defined in the EGM were least researched thematic areas in the literature. However, the findings in the EGM have implications for future research and policy decision‐making. Previous studies have highlighted the effects on mobile phone and radio on the rate of smallholder farmers' adoption of improved agriculture technology in SSA (Hudson et al. 2017). Consequently, farm households with access to information have increased in agricultural productivity and dietary diversity (Mwalupaso et al. 2020). Similarly, access to information and communication empowers women to take right decision on their economic situations (Rimi and Chudi 2017).

It was observed that more than half of the studies in the EGM were impact evaluations, and about a third were summative evaluations. However, most of the studies employed non‐experimental study designs (i.e., non‐randomized studies). None of the studies used an experimental study design with a randomized control trial. Studies with qualitative methods, scoping reviews, and other literature reviews were included in the EGM to increase the chance of including eligible studies in the EGM. Also, the nuances of infrastructure's impact on women's economic empowerment and gender quality were best studied with qualitative methods. Potential biases are likely to be greater for non‐randomized studies compared with randomized trials when evaluating the effects of interventions (Reeves et al. 2019). This implies future systematic reviews and meta‐analysis results should be interpreted with caution if they include non‐randomized studies as found in the EGM (Reeves et al. 2019). Future primary studies should consider using robust experimental study designs to evaluate the effects of infrastructure on nutritious diet, women's economic empowerment, and gender equality.

Five uncompleted studies on the effects of infrastructure on nutritious diet, women's economic empowerment, and gender equality, commissioned by ICED are ongoing in Ghana, Nigeria, and Ethiopia in the sub‐Saharan African region to fill in some of the evidence gaps identified in the EGM. These ongoing studies are focused on the impact of information infrastructure, roads, food processing facilities, market, and storage facilities on nutritious diets and women's economic empowerment and gender equality. This EGM will be updated periodically as new evidence evolves and resources are available. This EGM provides an access platform with a body of evidence on the typology of infrastructure relevant for agricultural and local economic development with effects on nutritious diet, women's economic empowerment, and gender equality.

#### 4.4.3 Potential Biases in the Mapping Process

##### 4.4.3.1 Strengths

A map on physical infrastructure's impact on nutritious diet, women's economic empowerment, and gender equality is novel. To the best of our knowledge, this is the first EGM on this topic. Few existing EGMs addressed food systems and gender equality in agriculture, but did not relate the various outcomes of nutritious diets, women's economic empowerment, and gender equality with physical infrastructure (LEAD 2021). This map contains 342 studies that were published or made available between 2000 and 2023. The map provides the most recent and available evidence on the topic for evidence‐based decision‐making. As much as possible, we used a comprehensive and exhaustive search strategy to identify the eligible studies. The search strategy was supplemented with the search for eligible studies in the three academic databases with OpenAlex machine learning searches. The use of OpenAlex machine learning in EPPI‐Reviewer data management software offered an opportunity for a comprehensive and exhaustive search for eligible studies. Given that OpenAlex is a literature source indexed with hundreds of millions of interconnected entities across the global research system (Priem et al. 2022), this helped to minimize selection bias in the identification of eligible studies for the EGM. Publication bias in this EGM was reduced by searching in academic databases, OpenAlex, as well as gray literature sources, including relevant organizational websites. This allowed us to include published and unpublished literature, as well as ongoing studies and completed studies in the EGM.

Priority screening using machine learning in EPPI‐Reviewer data management software of the title and abstract allowed us to screen the most relevant studies by single screening, while the full‐text screening was done by two independent reviewers. This ensured that all eligible papers were included in the EGM to avoid selection bias. Backward citations of included systematic reviews and other reviews, hand‐searches, as well as contacting key authors, were conducted to minimize selection bias.

#### 4.4.4 Limitations of the EGM

*Year of publication:* Limiting the year of publication to 2000 and beyond could be a source of selection bias in the EGM. Year of publication restriction was applied in this EGM to ensure identified studies reflected current evidence relevant to the ongoing discourse on infrastructural interventions' impact on nutritious diet, women's economic empowerment, and gender equality essential for achieving the SDGs. However, this publication date restriction may have inadvertently eliminated important studies and historic evidence on some of the interventions and outcome categories, resulting in sparse evidence for some of the thematic areas.

*English language restriction:* Studies written in non‐English languages were excluded from this EGM, which may have introduced selection bias. This was because most researchers were only English‐speaking, and including publications in a non‐English language would require additional resources for translation (Neimann Rasmussen and Montgomery 2018). However, the effect of this bias could be minimal because literature shows that evidence synthesis is not affected when the effect sizes from non‐English studies are excluded from analysis (Higgins et al. 2019).

##### 4.4.4.1 Stakeholder Engagement Throughout the EGM Process

There was no change in stakeholder engagement throughout the EGM process. The authors worked in close consultation with the Campbell Collaboration to produce this EGM.

## 5 Authors Conclusion

This EGM enhances the visibility and accessibility of the existing body of evidence on the typology of infrastructure investments that support a nutritious diet, women's economic empowerment, and gender equality outcomes for evidence‐based decision‐making in one‐spot for potential research commissioners, funders, policy makers, and researchers. The evidence in the EGM is mostly from impact and summative evaluations drawn from non‐experimental studies. The evidence spans a period of over two decades and covers 54 countries across SSA and SA regions. Eastern Africa region is the most studied population, and production infrastructure, specifically irrigation, was the most studied intervention. Central African region is the least studied population in the EGM. Overall, nutritious diets outcomes are well‐researched, while women's economic empowerment and gender equality outcomes are moderately researched. The current EGM has 337 completed and 5 ongoing studies.

### 5.1 Implications for Research, Practice, and/or Policy

- This EGM underscores a huge evidence gap on systematic reviews and meta‐analysis on physical infrastructure. There are also no effectiveness studies (randomized control trials) on infrastructure to inform decision‐making on the impact of intervention on nutritious diet, women's economic empowerment, and gender equality outcomes. This presents a significant challenge for policymakers implementing evidence‐informed interventions, given the absence of such studies providing high‐quality evidence. However, we must acknowledge that the complexity and contextual variability of empowerment and gender equality may make it difficult to design rigorous experimental studies or meta‐analyses. In this respect, we recommend consideration of the suitability and feasibility of these designs by underlining that future studies should focus on other, contextually appropriate methodologies able to provide better capturing of intricacies in these outcomes. This nuancing will serve to provide a deeper insight into the best methodology that the researcher and practitioner can use in investigating infrastructure on gender‐related outcomes.
- Production infrastructure and nutritious diets, and post‐production infrastructure and nutritious diets were well‐researched areas with more than 75 studies. The cluster of evidence identified in thematic areas should inform decision‐making on future evidence synthesis and infrastructure investment useful for sustainable nutrition and food systems, women's economic empowerment, and gender equality. Most of the research focus on these areas are solid ground for future exploration and development into policy level. This kind of evidence allows policymakers to identify successful interventions and scale them up in similar contexts. For instance, putting investment into irrigation systems or developing storage facilities may be prioritized based on their effectiveness in increasing food security and improving dietary quality. Additionally, the focus on these areas will allow meta‐analyses and systematic reviews that integrate findings from multiple studies to be undertaken in the future, providing greater insights into how infrastructure influences food systems and gender outcomes. Such an evidence base can also direct investments in technology and innovation to industrialize infrastructure for improved nutrition impact.
- Generally, production infrastructure and women's economic empowerment, as well as post‐production and women's economic empowerment, were moderately supported by evidence (25–75 studies). This suggests some level of understanding but highlights the need for deeper exploration, especially in varied contexts. Distribution and information infrastructure were the most under‐researched thematic areas (less than 25 studies). Resources should be allocated to these under‐researched thematic areas for more primary studies and evidence synthesis. Placing greater emphasis on these physical infrastructure types may help address important knowledge gaps and facilitate more integrated infrastructure approaches, yielding comprehensive benefits for food systems, gender equality, and economic opportunities.
- The EGM highlight types of infrastructure that have influence on nutritious diets, women's economic empowerment and gender equality, and informs advocacy for the scaling up of infrastructural interventions for equitable livelihoods, gender equality and women's economic empowerment.

Finally, the EGM can serve as a useful tool to incentivize governments and other donors to prioritize investments in infrastructure to improve the ability of agricultural and food systems to make nutritious diets affordable, accessible, and safe for LICs and subsequently empower women. Additionally, this EGM could inform governments and donors on how to design infrastructural intervention to maximize their impact on nutritious diet, women's economic empowerment and gender equality.

## Author Contributions

*Content*

- The Principal Investigator of the EGM, David Sarfo Ameyaw (PhD) is the CEO/President of ICED. He is a content expert in food security and infrastructural projects in developing countries. He has over 30 years of experience in leadership and practical experience in international development, monitoring and evaluation, learning, research, and EGM.
- Takyiwaa Manuh (PhD), the gender specialist and a distinguished University Professor, Emerita, is a subject matter expert in women's empowerment and gender equality. She has several scholarly publications on women's economic empowerment and gender equality and is skillful in policy dialog on women and gender issues in Ghana and beyond.
- Charles Yaw Okyere (PhD) is a Senior Lecturer at the Department of Agricultural Economics and Agribusiness, University of Ghana, Legon, and a Research Associate at ICED. Charles Yaw Okyere holds a Doctor of Agricultural Sciences (Dr. Agr.) degree from the University of Bonn, Germany. He has expertise skills in generating rigorous evidence for policy making through applying behavioral, experimental, and quasi‐experimental economic techniques to agriculture, health, education, and welfare.
- Solomon Zena Walelign (PhD), the research director of ICED, is an experienced environmental and resource economist with expertise in forest sciences. He is also a consultant at the World Bank and an Adjunct Assistant Professor at the University of Gondar. His previous research experience from Nepal, Ethiopia, Kenya, and Tanzania to the team.
- Gloria Odei Obeng‐Amoako (PhD), a nutrition and food systems specialist with ICED, has extensive experience in nutrition, public health, and epidemiology programming and research. She is currently an adjunct lecturer at the Department of Nutrition and Food Science, Biological Sciences, University of Ghana.
- Ms. Clarice Panyin Nyan (PhD) is a candidate at the Regional Institute for Population Studies (RIPS), University of Ghana, and also a Research and Evidence Synthesis Fellow at ICED with experience in evidence synthesis.

*EGM Methods*

- Ms. Clarice Panyin Nyan, Mr. Edward Kusi Asafo‐Agyei, Joseph Clottey (PhD), and Sheila Agyemang Oppong (PhD) at ICED did most of the screening and coding of the eligible studies in EPPI‐Reviewer Data Management software.
- Gloria Odei Obeng‐Amoako coordinated the daily EGM‐related activities to ensure smooth implementation of the protocol and the production of the EGM.
- Solomon Zena Walelign, Takyiwaa Manuh, and Charles Okyere reviewed and validated studies included in the EGM.
- Gloria Odei Obeng‐Amoako analyzed the EGM data and interpreted the results. Gloria Odei Obeng‐Amoako, Clarice Panyin Nyan, and Solomon Zena Walelign wrote the first draft of the manuscript. All the co‐authors read and reviewed the manuscript for publication.
- The team received technical backstopping from Drs. Howard White of the Campbell Collaboration, Ashrita Saran and Suchi Malhotra of the Campbell Collaboration Asia. All renowned EGM experts with several years of experience in EGM methodology.

*Information Retrieval*

- Information retrieval was conducted by Mr. Rodney Malesi, an experienced Senior Librarian affiliated with the United States International University, Kenya. Mr. Malesi is an astute librarian and expert in literature retrieval and has been involved in several systematic reviews and EGMs. Clarice Panyin Nyan led the team to retrieve eligible studies from gray literature sources.

## Conflicts of Interest

The authors declare no conflicts of interest.

## Preliminary Timeframe

Approximate date for submission of the EGM: September 2023.

## Plans for Updating the EGM

ICED will update the EGM on a bi‐annual basis with emerging evidence and as funding becomes available.

## Link to Online Interactive EGM

https://products.iced-eval.org/egm-iindwege.html.

## Supporting information

Supplementary_results_1__Frequency_of_countries.

Supplementary_results_2_Distribution_of_intervention_sub‐_categories_and_outcome_sub‐categories.

Supplementary_results_3__interventions_and_countries.

Supplementary_results_4__Outcomes_and_countries.

Supplementary_results_5_Distribution_of_intervention_sub‐categories_by_countries_in_the_included_studies.

Supplementary_results_6__Outcome_sub‐categories_and_countries.

supmat.
