# Supplementary material for: The Impact of Infrastructure on Low‐Income Consumers' Nutritious Diet, Women's Economic Empowerment, and Gender Equality in Low‐ and Middle‐Income Countries: An Evidence and Gap Map
Source: Campbell Syst Rev. 2025 Jul 18;21(3):e70050. doi: 10.1002/cl2.70050 (PMC12272306; doi:10.1002/cl2.70050)
Supplement: Supplementary file 7 — supmat. [file CL2-21-e70050-s003.docx]

**Appendices**

**Appendix A. Search Strategy for Websites Including Google and Google Scholar**

|  | **IINDWEGE Project** | | | |
| --- | --- | --- | --- | --- |
|  | **REPORTING SEARCH STRATEGY _EGM** | | | |
|  | **Accessing papers from websites** | | | |
| **No.** | **Data accesses/ searched** | **Source (Name/ URLs)** | **Search term** | **Number of hits (results)** |
| 1 | 6/27/2022 | **NBER (https://www.nber.org/)** | "nutritious diet" | 25 |
| 2 | 6/27/2022 |  | "women's economic empowerment" | 0 |
| 3 | 6/27/2022 |  | "gender equality" | 280 |
| 4 | 6/27/2022 | **EBSCO-Open Dissertations (https://ebsco.com/products/research-databases/ebsco-open-dissertations)** | "nutritious diet" | 16 |
| 5 | 6/27/2022 |  | "women's economic empowerment" | 12 |
| 6 | 6/28/2022 |  | "gender equality" | 591 |
| 7 | 6/28/2022 | **Research for Development Output (DfID) (https://www.gov.uk/research-for-development-outputs)** | "nutritious diet" | 3 |
| 8 | 6/28/2022 |  | "women's economic empowerment" | 117 |
| 9 | 6/28/2022 |  | "gender equality" | 215 |
| 10 | 7/6/2022 | **International Food Policy Research Institute (IFPRI) (https://www.ifpri.org/)** | "nutritious diet" | 57 |
| 11 | 7/6/2022 |  | "women's economic empowerment" | 56 |
| 12 | 7/6/2022 |  | "irrigation and women empowerment" | 22 |
| 13 | 7/6/2022 |  | "gender equality" | 22 |
| 14 | 7/6/2022 |  | "infrastructure" | 91 |
| 15 | 6/27/2022 | **Agriculture, Nutrition and Health (ANH) Academy conference, (IMMANA-inclusive) (https://www.anh-academy.org/)** | "nutritious diet" | 69 |
| 16 | 6/27/2022 |  | "women's economic empowerment" | 23 |
| 17 | 6/27/2022 |  | "gender equality" | 6 |
| 18 | 7/12/2022 | **International Water Management Institute (IWMI), World Bank (https://www.iwmi.cgiar.org/)** | "nutritious diet" | 4 |
| 19 | 7/12/2022 |  | "women's economic empowerment" | 0 |
| 20 | 7/12/2022 |  | "women's empowerment" | 7 |
| 21 | 7/12/2022 |  | "gender equality" | 59 |
| 22 | 7/13/2022 |  | "infrastructure" | 394 |
| 23 | 7/13/2022 |  | impact of infrastructure on nutritious diet | 8 |
| 24 | 7/13/2022 |  | impact of infrastructure on women economic empowerment | 34 |
| 25 | 7/13/2022 |  | impact of infrastructure on gender equality | 51 |
| 26 | 7/15/2022 | **Donor Committee for Enterprise Development (DCED) (https://www.enterprise-development.org/)** | nutritious diet | 0 |
| 27 | 7/15/2022 |  | nutrition | 5 |
| 28 | 7/15/2022 |  | women's economic empowerment | 26 |
| 29 | 7/15/2022 |  | women empowerment | 38 |
| 30 | 7/15/2022 |  | gender equality | 10 |
| 31 | 7/15/2022 |  | impact of infrastructure on nutritious diet | 0 |
| 32 | 7/15/2022 |  | impact of infrastructure on women economic empowerment | 7 |
| 33 | 7/15/2022 |  | impact of infrastructure on gender equality | 3 |
| 34 | 7/18/2022 | **Alliance for a Green Revolution in Africa (AGRA) (https://agra.org/)** | "nutritious diet" | 2 |
| 35 | 7/18/2022 |  | "nutrition" | 61 |
| 36 | 7/18/2022 |  | "women's economic empowerment" | 0 |
| 37 | 7/18/2022 |  | "women empowerment" | 9 |
| 38 | 7/18/2022 |  | "gender equality" | 3 |
| 39 | 7/18/2022 |  | impact of infrastructure on nutritious diet | 0 |
| 40 | 7/18/2022 |  | impact of infrastructure on women economic empowerment | 1 |
| 41 | 7/18/2022 |  | impact of infrastructure on gender equality | 0 |
| 42 | 7/18/2022 |  | "infrastructure" | 49 |
| 43 | 7/18/2022 | **International Institute for Environment and Development (IIED) (https://www.iied.org/)** | nutritious diet | 38 |
| 44 | 7/18/2022 |  | nutrition | 70 |
| 45 | 7/19/2022 |  | women's economic empowerment | 1566 |
| 46 | 7/19/2022 |  | infrastructure | 175 |
| 47 | 7/19/2022 |  | gender equality | 334 |
| 48 | 7/19/2022 | **The Journal of Electronic Theses and Dissertations (J-ETD) (https://ndltd.org/etd-journal/)** | nutritious diet | 0 |
| 49 | 7/19/2022 |  | nutrition | 0 |
| 50 | 7/19/2022 |  | women's economic empowerment | 0 |
| 51 | 7/19/2022 |  | women empowerment | 0 |
| 52 | 7/19/2022 |  | gender equality | 0 |
| 53 | 7/19/2022 |  | impact of infrastructure on nutritious diet | 0 |
| 54 | 7/19/2022 |  | impact of infrastructure on women economic empowerment | 0 |
| 55 | 7/19/2022 |  | impact of infrastructure on gender equality | 0 |
| 56 | 7/19/2022 |  | infrastructure | 0 |
| 57 | 7/23/2022 | **International Fund for Agric Development (IFAD) (https://www.ifad.org/en/)** | "nutritious diet" | 28 |
| 58 | 7/23/2022 |  | "women's economic empowerment" | 34 |
| 59 | 7/23/2022 |  | "women empowerment" | 210 |
| 60 | 7/23/2022 |  | "gender equality" | 293 |
| 61 | 7/23/2022 |  | impact of infrastructure on nutritious diet | 0 |
| 62 | 7/23/2022 |  | impact of infrastructure on women economic empowerment | 0 |
| 63 | 7/23/2022 |  | impact of infrastructure on gender equality | 0 |
| 64 | 7/24/2022 |  | "infrastructure" | 1107 |
| 65 | 7/25/2022 | **Organisation for Economic Planning and Development/Development Planning Committee (DAC-OECD) (https://www.oecd.org/en/about/committees/development-assistance-committee.html)** | "nutritious diet" | 0 |
| 66 | 7/25/2022 |  | "nutrition" | 30 |
| 67 | 7/25/2022 |  | "women's economic empowerment" | 17 |
| 68 | 7/25/2022 |  | "women empowerment" | 58 |
| 69 | 7/25/2022 |  | "gender equality" | 306 |
| 70 | 7/25/2022 |  | "impact of infrastructure on nutritious diet" | 0 |
| 71 | 7/25/2022 |  | "impact of infrastructure on women economic empowerment" | 0 |
| 72 | 7/25/2022 |  | "impact of infrastructure on gender equality" | 0 |
| 73 | 7/25/2022 |  | "infrastructure" | 859 |
| 74 | 6/21/2022 | **J-PAL (https://www.povertyactionlab.org/)** | "nutritious diet" | 0 |
| 75 | 6/21/2022 |  | "women's economic empowerment" | 6 |
| 76 | 6/21/2022 |  | "gender equality" | 63 |
| 77 | 6/21/2022 | **Independent Evaluations Group (IEG)- World Bank (https://ieg.worldbankgroup.org/evaluations)** | "nutritious diet" | 0 |
| 78 | 6/21/2022 |  | "women's economic empowerment" | 0 |
| 79 | 6/21/2022 |  | "gender equality" | 50 |
| 80 | 6/22/2022 | **Food and Agriculture Organization of the United Nations- Agricultural Science and Technology Information (FAO-AGRIS) (https://agris.fao.org/)** | "nutritious diet" | 39 |
| 81 | 6/22/2022 |  | "women's economic empowerment" | 22 |
| 82 | 6/22/2022 |  | "gender equality" | 1062 |
| 83 | **7/15/2022** | **Development Experience Clearinghouse (DEC)- USAID (https://dec.usaid.gov/dec/)** | "agricultural infrastructure" | 100 |
| 84 | 7/15/2022 |  | "agricultural infrastructure" | 292 |
| 85 | 7/15/2022 |  | "agricultural infrastructure" | 14 |
| 86 | 7/15/2022 |  | "agricultural infrastructure" | 92 |
| 87 | 7/15/2022 |  | "agricultural infrastructure" | 414 |
| 88 | 7/15/2022 |  | "nutritious diet" | 0 |
| 89 | 7/15/2022 |  | "gender equality" | 458 |
| 90 | 7/17/2022 | **Swiss Agency for Dev't and Coop. (SDC) (https://www.swissaid.ch/en/swiss-agency-for-development-and-cooperation/)** | "nutritious diet" | 0 |
| 91 | 7/17/2022 |  | "women's economic empowerment" | 0 |
| 92 | 7/17/2022 |  | "gender equality" | 0 |
| 93 | 7/17/2022 |  | "agricultural infrastructure" | 0 |
| 94 | 7/17/2022 |  | "infrastructure" | 100 |
| 95 | 7/17/2022 |  | "healthy diet" | 7 |
| 96 | 7/17/2022 |  | "irrigation" | 0 |
| 97 | 7/17/2022 |  | "impact of roads on women's economic empowerment" | 0 |
| 98 | 7/17/2022 |  | "road infrastructure improves food security" | 0 |
| 99 | 7/17/2022 |  | "food availability" | 13 |
| 100 | 7/17/2022 |  | "women's empowerment" | 0 |
| 101 | 7/17/2022 |  | "women and agriculture" | 0 |
| 102 | 7/17/2022 |  | "Women's Empowerment in Agriculture index" | 0 |
| 103 | 7/25/2022 | **Google (https://www.google.com.gh/)** | "infrastructure impact on nutritious diet" | 6,050,000 |
| 104 | 7/25/2022 |  | infrastructure impact on women's economic | 145,000,000 |
| 105 | 7/25/2022 |  | impact of infrastructure on gender equality | 16,600,000 |
| 106 | 7/26/2022 | **Google scholar (https://scholar.google.com/)** | infrastructure's impact on nutritious diet | 11 |
| 107 | 7/26/2022 |  | infrastructure's impact on nutritious diet | 24,000 (screened 1^st^ 8 pages) |
| 108 | 7/26/2022 |  | infrastructure's impact on women's economic empowerment | 17,900 (screened 1^st^ 4 page) |
| 109 | 7/26/2022 |  | infrastructure's impact on gender equality | 20,400 (screened 1^st^ 9 pages) |
| 110 | 7/4/2022 | **International Livestock Research Institute (ILRI) (https://www.ilri.org/)** | "infrastructure effect on nutritious diet" | 10 |
| 111 | 6/27/2022 |  | "women's economic empowerment" | 5 |
| 112 | 6/27/2022 |  | "women equality" | 1 |
| 113 | 6/27/2022 | **World Agroforestry** | "nutritious diet" | 21 |
| 114 | 7/3/2022 | **(https://www.worldagroforestry.org/about)** | "women's empowerment" | 45 |
| 115 | 7/3/2022 |  | "gender equality" | 168 |
| 116 | 7/4/2022 |  | "infrastructure" | 1646 |
| 117 | 7/4/2022 | **International Initiative for Impact Evaluation 3ie (https://www.3ieimpact.org/)** | "nutritious diet" | 22 |
| 118 | 7/4/2022 |  | "women's economic empowerment" | 502 |
| 119 | 7/4/2022 |  | "gender equality" | 189 |
| 120 | 6/27/2022 | **British Library for Development Studies (BLDS) (https://bldslegacycollection.uk/)** | "nutritious diet" | 357 |
| 121 | 6/27/2022 |  | "women's economic empowerment" | 2882 |
| 122 | 6/27/2022 |  | "gender equality" | 4978 |
| 123 | 7/4/2022 | **Innovation for Poverty Action (IPA) (https://poverty-action.org/)** | "nutritious diet" | 1 |
| 124 | 7/4/2022 |  | "women's economic empowerment" | 86 |
| 125 | 7/4/2022 |  | "gender equality" | 29 |
| 126 | 7/4/2022 |  | "diet" | 16 |
| 127 | 7/12/2022 | **UN Women (https://www.unwomen.org/en)** | "nutrition" | 174 |
| 128 | 7/12/2022 |  | "women's economic empowerment" | 2548 |
| 129 | 7/12/2022 |  | "gender equality" | 4796 |
| 130 | 7/12/2022 |  | "diet" |  |
| 131 | 7/18/2022 | **FAO (https://www.fao.org/home/en)** | "nutritious diet" on webpage | 29,900 |
| 132 | 7/18/2022 |  | "women economic empowerment" on webpage | 31,200 |
| 133 | 7/18/2022 |  | "gender norm" | 42,100 |
| 134 | 7/18/2022 |  | "dietary diversity" | 20,500 |
| 135 | 7/18/2022 |  | "nutritious diet" on publication page | 275 |
| 136 | 7/18/2022 |  | "women empowerment" on publication page | 6660 |
| 137 | 7/18/2022 |  | gender | 9590 |
| 138 | 7/19/2022 | **BMGF (https://www.gatesfoundation.org/)** | "nutritious diet" on webpage | 12 |
| 139 | 7/19/2022 |  | "women economic empowerment" | 11 |
| 140 | 7/21/2022 |  | "gender equality" | 1435 |
| 141 | 7/22/2022 |  | "dietary diversity" on webpage | 4 |
| 142 | 7/19/2022 |  | household food on webpage | 51 |
| 143 | 7/19/2022 | **CGIAR (https://www.cgiar.org/)** | "nutritious diet" | 127 |
| 144 | 7/19/2022 |  | "women's economic empowerment" | 164 |
| 145 | 7/19/2022 |  | "gender equality" | 52 |
| 146 | 7/19/2022 |  | "diet" | 177 |
| 147 | 7/19/2022 |  | "nutritious food'' | 16 |
| 148 | 7/19/2022 | **Biodiversity Heritage Library (https://www.biodiversitylibrary.org/)** | "nutritious diet 2000 or later" | 332 |
| 149 | 7/19/2022 |  | "women empowerment" | 136 |
| 150 | 7/19/2022 |  | "gender equality articles" | 33 |
| 151 | 7/13/2022 | **AgriProFocus (https://www.snv.org/project/agriprofocus-apf-making-agribusiness-work-development-0)** | "nutritious diet" | ERROR |
| 152 | 7/13/2022 |  | "women's economic empowerment" | ERROR |
| 153 | 7/13/2022 |  | "gender equality" | ERROR |
| 154 | 7/13/2022 |  | "diet" | ERROR |
| 155 | 7/25/2022 | **Campbell Collaboration (https://www.campbellcollaboration.org/)** | "nutritious diet" | 1 |
| 156 | 7/25/2022 |  | "women empowerment" | 14 |
| 157 | 7/25/2022 |  | "gender equality" | 6 |
| 158 | 7/25/2022 |  | "women leadership" | 5 |
| 159 | 7/25/2022 | **Campbell South Asia (https://campbellsouthasia.org/)** | "transport intervention" | 4 |
| 160 | 7/25/2022 |  | "infrastructure interventions" | 2 |
| 161 | 7/25/2022 |  | "gender equality" | 3 |
| 162 | 7/25/2022 |  | "diet" | 0 |
| 163 | 7/29/2022 | **World Bank Economic Review (https://documents.worldbank.org/en/publication/documents-reports/documentdetail/492231565381915827/the-world-bank-economic-review-31-2)** | "nutritious diet" | 2 |
| 164 | 7/29/2022 |  | "food markets" | 428 |
| 165 | 7/29/2022 |  | "dietary diversity" | 11 |
| 166 | 7/29/2022 |  | "Household Food Insecurity Access Scale" | 21 |
| 167 | 7/29/2022 |  | "women empowerment" | 32 |
| 168 | 7/29/2022 |  | "women leadership" | 26 |
| 169 | 7/29/2022 |  | "gender equality" | 268 |

**Appendix B: Search Strategy for Registries**

|  | | **REGISTRIES** | | | |
| --- | --- | --- | --- | --- | --- |
| **TITLE AND ABSTRACT SCREENING** | | | | | |
| **No.** | **Date accessed/ searched** | | **Source** | **Search term** | **Number of hits (results)** |
| 1 | 8/4/2022 | | **The Cochrane Central Register of Controlled Trials (CENTRAL)** | "nutritious diet" | 20 |
| 2 | 8/4/2022 | |  | "women empowerment" | 93 |
| 3 | 8/4/2022 | |  | "women economic empowerment" | 5 |
| 4 | 8/4/2022 | |  | "gender equality" | 64 |
| 5 | 8/5/2022 | | **American Economic Association (AEA)** | "nutritious diet" | 0 |
| 6 | 8/5/2022 | |  | "nutrition" | 0 |
| 7 | 8/5/2022 | |  | "women's economic empowerment" | 0 |
| 8 | 8/5/2022 | |  | "women empowerment" | 0 |
| 9 | 8/5/2022 | |  | "gender equality" | 0 |
| 10 | 8/5/2022 | |  | "impact of infrastructure on nutritious diet" | 0 |
| 11 | 8/5/2022 | |  | "impact of infrastructure on women economic empowerment" | 0 |
| 12 | 8/5/2022 | |  | "impact of infrastructure on gender equality" | 0 |
| 13 | 8/5/2022 | |  | "infrastructure" | 0 |
| 14 | 8/5/2022 | | **The Registry for International Development Impact Evaluations (RIDIE)** | "nutritious diet" | 246 |
| 15 | 8/5/2022 | |  | "women's economic empowerment" | 0 |
| 16 | 8/5/2022 | |  | "women empowerment" | 6 |
| 17 | 8/5/2022 | |  | "gender equality" | 20 |
| 18 | 8/5/2022 | |  | "infrastructure" | 50 |
| 19 | 8/5/2022 | |  | "impact of infrastructure on nutritious diet" | 0 |
| 20 | 8/5/2022 | |  | "impact of infrastructure on women economic empowerment" | 0 |
| 21 | 8/5/2022 | |  | "impact of infrastructure on gender equality" | 0 |
| 22 | 8/5/2022 | | **Campbell Library** | "nutritious diet" | 7535 |
| 23 | 8/5/2022 | |  | "food markets" | 94061 |
| 24 | 8/5/2022 | |  | "dietary diversity" | 20738 |
| 25 | 8/5/2022 | |  | "Household Food Insecurity Access Scale" | 322 |
| 26 | 8/5/2022 | |  | "women empowerment" | 2449 |
| 27 | 8/5/2022 | |  | "women leadership" | 802 |
| 28 | 8/5/2022 | |  | "gender equality" | 54198 |
| 29 | 8/5/2022 | | **Cochrane Library** | "nutritious diet" | 5 |
| 30 | 8/5/2022 | |  | "food markets" | 52 |
| 31 | 8/5/2022 | |  | "dietary diversity" | 4 |
| 32 | 8/5/2022 | |  | "Household Food Insecurity Access Scale" | 19 |
| 33 | 8/5/2022 | |  | "women empowerment" | 87 |
| 34 | 8/5/2022 | |  | "women leadership" | 0 |
| 35 | 8/5/2022 | |  | "gender equality" | 1 |
| 36 | 8/5/2022 | | **Database of Abstracts of Reviews of Effects (DARE)** | "nutritious diet" | 1 |
| 37 | 8/5/2022 | |  | "food markets" | 0 |
| 38 | 8/5/2022 | |  | "dietary diversity" | 0 |
| 39 | 8/5/2022 | |  | "Household Food Insecurity Access Scale" | 0 |
| 40 | 8/5/2022 | |  | "women empowerment" | 0 |
| 41 | 8/5/2022 | |  | "women leadership" | 0 |
| 42 | 8/5/2022 | |  | "gender equality" | 1 |
| 43 | 8/5/2022 | |  | "infrastructure" | 1 |
| 44 | 8/5/2022 | | **International Prospective Register of Systematic Reviews (PROSPERO)** | "nutritious diet" | 2 |
| 45 | 8/5/2022 | |  | "food markets" | 7 |
| 46 | 8/5/2022 | |  | "dietary diversity" | 81 |
| 47 | 8/5/2022 | |  | "Household Food Insecurity Access Scale" | 5 |
| 48 | 8/5/2022 | |  | "women empowerment" | 7 |
| 49 | 8/5/2022 | |  | "women leadership" | 2 |
| 50 | 8/5/2022 | |  | "gender equality" | 43 |
| 51 | 8/5/2022 | | **Journal for Development Economics (JDE)** | "nutritious diet" | 3 |
| 52 | 8/5/2022 | |  | "nutritious" | 13 |
| 53 | 8/5/2022 | |  | "women economic empowerment" | 5 |
| 54 | 8/5/2022 | |  | "gender equality" | 36 |
| 55 | 8/5/2022 | |  | "impact of infrastructure on nutritious diet" | 0 |
| 56 | 8/5/2022 | |  | "impact of infrastructure on women economic empowerment" | 0 |
| 57 | 8/5/2022 | |  | "impact of infrastructure on gender equity" | 0 |

**Appendix C: Search Strategy used for CAB Direct, PubMed and GreenFILE search**

| **Category** |  | **Search Terms (All Fields)** |
| --- | --- | --- |
| **1. Population** | **1**        LMIC _1 | ("Afghanistan" OR "Angola" OR "Bangladesh" OR "Benin" OR "Botswana" OR "Burundi" OR "Bhutan" OR "cabo verde" OR "Cape Verde" OR “Cap Vert” OR "cameroon" OR "central African republic "OR "ubangi shari" OR "chad" OR "tchad" OR "Comoros" OR "Comoro" OR "comoro islands "OR "democratic republic of the Congo" OR "zaire" OR “Congo Brazzaville” OR "cote d Ivoire" OR "Ivory Coast" OR "Djibouti" OR "French Somaliland" OR "Equatorial Guinea" OR "eritrea" OR "Ethiopia" OR "Ethiopia" OR "Eswatini" OR "Gabon" OR "Gabonese Republic" OR "Gambia" OR "Ghana" OR "Gold coast" OR "guinea" OR “Guinea Bissau” OR "India" OR "Iran" OR " OR "kenya" OR "lesotho" OR "Basutoland" OR "Liberia" OR "Liberia s" OR "madagascar" OR "Malagasy republic" OR "Malawi" OR "Nyasaland" OR "Mali" OR "Indian ocean islands" OR "Maldives" OR "maldive" OR "Mauritania" OR "Mauritius" OR "Mozambique" OR "Namibia" OR "Nepal" OR "Niger" OR "Nigeria" OR "Rwanda" OR "Ruanda" OR "sao tome and Principe" OR "Senegal" OR "Seychelles" OR "Sierra Leone" OR "Somalia" OR "south africa" OR "south Sudan" OR “Sudan” OR "Sri Lanka" OR "Pakistan" OR "Tanzania" OR "Tanganyika" OR "Togo" OR "Togolese republic" OR "Uganda" OR "Zambia" OR "Zimbabwe" OR "northern Rhodesia" OR "global south" OR "africa south of the sahara" OR "sub-Saharan Africa" OR "sub-Saharan Africa" OR "africa central" OR "central africa" OR "africa southern" OR "southern africa" OR "africa eastern" OR "east africa" OR "eastern africa" OR "africa western" OR "west africa" OR "western africa" OR "asia southern" OR "southern asia" OR "south asia") OR |
|  | **2**      LMIC_2 | (("developing country" OR "developing world" OR "less developed countries" OR "less developed populations" OR "less developed world" OR "under developed nations" OR "under developed populations" OR "under developed world" OR "underdeveloped countries" OR "underdeveloped populations" OR "middle income countries" OR "middle income nation" OR "middle income population" OR "low income countries" OR "low income nations" OR "low income populations" OR "middle income countries" OR "lower income nations" OR "lower income population" OR "developing countries" OR "developing nation" OR "underdeveloped nation" OR "third world countries" OR "emerging economies" OR "poor countries" OR "poor nations" OR "poor population" OR "poor world" OR "poorer countries" OR "poorer nations" OR "poorer population" OR "developing economies") AND (“income” OR “poverty” OR “underdevelopment” OR “growth”)) |
|  | **3**  LMIC _1 OR LMIC _2 | (#1 OR #2) |
| **2. Intervention** | **4**    **Infrastructure** | ("Agricultural infrastructure" OR "agriculture infrastructure" OR "agricultural technology" OR "agricultural economics" OR "Rural Infrastructure" OR “physical infrastructure” OR "therapeutic irrigation" OR "drinking water" OR "supplies" OR "equipment and supplies" OR "mechanization" OR "farms" OR "floriculture" OR "infrastructure" OR "infrastructures" OR "horticulture" OR "dairy") OR |
|  | **5**      **Production** | ("irrigation system*" OR "irrigation" OR "water well*" OR "green infrastructure*" OR "garden* infrastructure*" OR "Electrical infrastructure" OR "power supply" OR "energy supply" OR “solar” OR "solar energy" OR "solar power" OR "solar power irrigation" OR "solar energy irrigation" OR “electrification” OR “small-scale irrigation” OR “farm assets” OR "green infrastructures" OR "green revolution infrastructures" OR "agricultural mechanization" OR "agricultural machiner*" OR "pastoral* infrastructure" OR "agropastoral infrastructure" OR "agro-pastoral infrastructure" OR "ejido" OR “silvopastoral infrastructure" OR "farm infrastructure*" OR "producer*" OR "grower*" OR "agronomy infrastructure" OR "husbandry infrastructure" OR "aquacultur* infrastructure" OR "floricultur* infrastructure" OR "horticultur* infrastructure" OR "cultivat* infrastructure" OR "dairy infrastructure" OR "livestock infrastructure" OR "crop* infrastructure") OR |
|  | **6**    **Post-production** | ("Food Handling" OR "Food Storage" OR "Food Quality" OR "storage facilit*" OR "storage" OR "storehouse" OR "store" OR Warehouse* OR sheds OR "cold room*" OR "processing facilities" OR process* OR mill OR "grain mills" OR market OR space* OR "market place" OR "market stalls" OR "booth" OR "market lockups" OR toilets OR "lavatory" OR "bathroom" OR "sanitation" OR "hygiene" OR "sanitary facilities" OR "market sanitation" OR "market place toilet" OR "market toilets" OR slaughterhouse* OR "abattoir" OR butcher* OR "landing site*" OR "fishing sites" OR "livestock" OR "vaccination parks" OR "vaccination" OR "animal vaccination park" OR " market access") OR |
|  | **7**    **Distribution** | "roads" OR "bridges" OR "railways" OR "transportation" OR "transportation infrastructures" OR "emergency transportation" OR "sustainable transportation" OR "rural roads" OR "distribution infrastructure" OR “transport cost”) OR |
|  | **8**    **Information** | ("agricultural information" OR "agricultural information" OR "information centers" OR "agricultural information dissemination" OR "information sharing" OR "radio stations" OR "community radio" OR television OR "TV" OR "telecommunication" OR "telecommunication masts" OR “mast” OR "information centre*" OR "telecommunication masts" OR "community radios" OR "information infrastructure" OR "telecommunication infrastructure") |
| **Intervention**  **(All terms & Fields)** | **9** | (#4 OR #5 OR #6 OR #7 OR #8) |
| **3. Outcome** | **10**    **Nutritious diet** | "Nutrition" OR "Diet quality" OR "dietary diversity" OR "Food Security" OR "nutrition security" OR "food affordability" OR "food groups" OR "improved diet" OR "food agency" OR "food insecurity" OR " dietary energy supply" OR "Household Food" OR "Minimum Dietary Diversity" OR "Mean adequacy ratio" OR "food price" OR "food price index" OR “nutritious diet” OR “Balanced diet” OR “Food availability” OR “food accessibility” OR “Food consumption” OR “Food production” OR “Food expenditure” OR “Household Income” OR “Food markets” OR “Retail Food” OR “fresh food” OR “Food groups” OR “Food supply” OR “Food demand” OR “food seasonality” OR “Food Insecurity Experience Scale” OR “FIES” OR “Household Food Insecurity Access Scale” OR “HFIAS” OR “household hunger scale” OR “HHS” OR “Women and nutrition” OR “Women and food” OR “household dietary diversity score” or “nutritious foods” OR “food insecure” OR “diverse nutritious foods” OR |
|  | **11** | (#3 AND #9) OR #10 |
|  | **12**    **Women’s economic empowerment** | "Women empowerment" OR "Social outcome" OR "resource allocation" OR "women employment" OR “female employment” OR “employment” OR “microenterprises” OR “ occupational choices” OR "asset ownership" OR "land access" OR "women leadership" OR "agricultural inputs" OR " agricultural technologies" OR "agricultural information access" OR "agricultural skill" OR "agriculture economic outcomes" OR "increase in income" OR "increase in farm investment" OR "resource use efficiency" OR "household assets" OR "healthy livestock" OR " time management" OR "social change" OR "social norms" OR "community leadership" OR "agricultural practices" OR "dissemination of knowledge" OR "crop information" OR "sustainable production practices" OR "gender equity" OR "agricultural value chain" OR "women in agricultural entrepreneurship" OR "livestock asset" OR “agency” OR “ women’s empowerment” OR “ female labour participation” OR “nonfarm employment” |
|  | 13 | (#3 AND #9) OR #12 |
|  | **14**    **Gender Equality** | ("Gender Equity" OR "gender equality" OR "gender parity" OR "gender inequality" OR "gender disparity" OR " gender norm" OR "gender mainstreaming" OR "gender" OR “gender roles” OR gender values” OR “gender needs”) |
|  | **15** | (#3AND #9) OR #14 |
| **Population, Interventions and Outcome**  (All terms & Fields) | **16 (Final combination)** | (#11 AND #13 AND #15) |

**Appendix D.** **Search Results**

| **Database** | **Category** |  | TOTAL SEARCH RESULTS | | | |
| --- | --- | --- | --- | --- | --- | --- |
|  |  |  | PubMed | GreenFILE | Cab Direct | GoogleScholar |
| PubMed |  | LMIC _1 |  |  |  | Combined Searches    861 |
|  | **1. Population** |  | 1,360,257 | 17,200 | 2,315,715 |  |
|  |  | LMIC_2 | 66782 | 17,800 | 3,371,728 |  |
|  |  | 3 LMIC _1 OR LMIC _2 |  | 96,555 | 2,856,644 |  |
|  | **2. Intervention** | **4 Infrastructure** | 258,812 | 55,875 | 1,734,733 |  |
|  |  | **5 Production** | 316716 | 71,604 | 505,623 |  |
|  |  | **6 Post- production** | 4860115 | 295,197 | 2,982,030 |  |
|  |  | **7 Distribution** | 407195 | 20,766 | 439,906 |  |
|  |  | **8 Information** | 324796 | 4,909 | 59,996 |  |
|  | **9 (All terms & Fields)** | **9** | 3,772,905 | 377,103 | 4,701,130 |  |
|  |  | **10 Nutritious diet** | 2,628,148 | 26,527 | 1,012,283 |  |
|  | **3. Outcome** | **11 (#3 AND #9) AND #10** | 28,563 | 64,215 | 137,485 |  |
|  |  | **12** | 319,093 | 53,270 | 243,889 |  |
|  |  | **Women’s economic empowerment** |  |  |  |  |
|  |  | 13 | 13,359 | 91,559 | 69,359 |  |
|  |  | **14** | 121,616 | 4,800 | 121,616 |  |
|  |  | **15(#3AND #9) OR #14** | 5576 | 44,260 | 19202 |  |
|  | **Population, Interventions and Outcome** | **16 (Final combination)** | 2119 | 3,771 | 8383 |  |

**Reference Management RIS Uploads and De-Duplication**

Total Uploaded on Mendeley Reference Management Software 13887

Total de-duplicated 60

Further citations older than Year 2000 2032

Total RIS citations generated and sent to EPPI 11795

| **Appendix D:. Papers Shared by Key Authors** | | |
| --- | --- | --- |
| Number | Name of author | Number of papers |
| 1 | Agnes Quisumbing | 2 |
| 2 | Franklin Amuakwa-Mensah | 1 |
| 3 | Jennifer Burney | 24 |

**Appendix E:. Coding Form**

| **Grandparent code** | **Parent code** | **Child code** | **Definition/examples** |
| --- | --- | --- | --- |
| ***Population (Low- and Middle-Income Countries)*** | **Regions (SSA and SA)** | **Countries** |  |
|  | **Central Africa** | Angola |  |
|  |  | Burundi |  |
|  |  | The Central African Republic |  |
|  |  | Chad |  |
|  |  | Democratic Republic of Congo |  |
|  |  | Congo |  |
|  |  | Rwanda |  |
|  | **Eastern Africa** | Comoros |  |
|  |  | Eritrea |  |
|  |  | Ethiopia |  |
|  |  | Kenya |  |
|  |  | Madagascar |  |
|  |  | Somalia |  |
|  |  | South Sudan |  |
|  |  | Sudan |  |
|  |  | Tanzania |  |
|  |  | Uganda |  |
|  | **Southern Africa** | Botswana |  |
|  |  | Eswatini |  |
|  |  | Lesotho |  |
|  |  | Malawi |  |
|  |  | Mozambique |  |
|  |  | Namibia |  |
|  |  | South Africa |  |
|  |  | Zambia |  |
|  |  | Zimbabwe |  |
|  | **Western Africa** | Benin |  |
|  |  | Burkina Faso |  |
|  |  | Cape Verde |  |
|  |  | Cameroon |  |
|  |  | Côte d'Ivoire |  |
|  |  | Equatorial Guinea |  |
|  |  | Gabon |  |
|  |  | The Gambia |  |
|  |  | Ghana |  |
|  |  | Guinea |  |
|  |  | Guinea-Bissau |  |
|  |  | Liberia |  |
|  |  | Mali |  |
|  |  | Mauritania |  |
|  |  | Niger |  |
|  |  | Nigeria |  |
|  |  | Sao Tome and Principe |  |
|  |  | Senegal |  |
|  |  | Sierra Leone |  |
|  |  | Togo |  |
|  | **South Asia** |  |  |
|  |  | Afghanistan |  |
|  |  | Bangladesh |  |
|  |  | Bhutan |  |
|  |  | India |  |
|  |  | Pakistan |  |
|  |  | Sri Lanka |  |
|  |  | Maldives |  |
|  |  | Nepal |  |
| ***Interventions*** | **Production infrastructure** | Irrigation systems and water wells | Irrigation system is an arrangement by which water is conveyed from a source to an area needing water to facilitate the production of desired crops (Kelly W.W, 1983)  Irrigation is the process to apply water to the soil to improve the crop growing, maintain landscapes and revegetate degraded soils in dry regions and periods of insufficient rainfall (Jimenez et al 2020). A well is a hole drilled into the ground to access water from an aquifer. A pipe and a pump are used to pull water out of the ground, and a screen filters out unwanted particles that could clog the pipe. Source: (Groundwater, 2022) Accessed in July 2022. https://www.groundwater.org/get-informed/basics/wells.html |
|  |  | Gardens and other green infrastructure | Green infrastructure is an emerging planning and design concept that is principally structured by a hybrid hydrological/drainage network, complementing, and linking relict green areas with built infrastructure that provides ecological functions (Ahern, J., 2007). |
|  |  | On-farm energy and power supply (solar, wind, water) | Energy and power supply used on farm for agricultural production. Energy and power supply (solar, wind, water) that used in directly in agricultural production (e.g. solar power infrastructure used for an irrigation). |
|  | **Post-production infrastructure** | Market facilities (sheds, market space, stalls with toilets, childcare centres | A market stall is a booth or stand where individuals or small businesses sell goods. These can range from artisan produce to food, antiques, clothes and much more. Markets can be in the form of a permanent fixture, as well as open-air markets, farmers markets, street markets and even car-boot sales. Source: https://squareup.com/au/en/townsquare/how-to-set-up-market-stall |
|  |  | Processing (grain mills) | Milling is the process of cleaning, tempering, and grinding cereal grains into flour and other milled grain products. Ground grain was one of civilization’s first foods. Source: https://namamillers.org/consumer-resources/what-is-milling/ |
|  |  | Storage (Cold room, warehouses and sheds) | Storage is an important marketing function, which involves holding and preserving goods from the time they are produced until they are needed for consumption. The storage of goods, therefore, from the time of production to the time of consumption, ensures a continuous flow of goods in the market. It protects the quality of perishable and semi-perishable products from deterioration; helps in the stabilization of prices by adjusting demand and supply. Storage also provides employment and income through price advantages. Source: (AGRITECH, 2022) https://agritech.tnau.ac.in/agricultural_marketing/agrimark_storage%20and%20ware%20housing.html#:~:text=Storage%20is%20an%20important%20marketing,of%20goods%20in%20the%20market. |
|  |  | Off-farm energy and power supply (solar, wind, water) | Energy and power supply used off-farm for post- production such as processing, storage etc. This includes indirect energy refers to the energy used to produce agricultural inputs. These inputs account for energy use that can be assigned to the agricultural sector but is used prior to reaching farms, including energy used in the: • _Production of fertilizers (raw materials, manufacturing, transport) • _Production of pesticides (raw materials, manufacturing, transport) • _Production, storage, and transportation of seeding materials. Direct energy refers to all energy inputs used directly in the agricultural production process; activities occurring on-farm and up to the farm gate (Paris et al 2022) |
|  |  | Livestock facilities (slaughterhouse,landing sites) | A slaughterhouse or abattoir or meat works is a facility where animals are killed for consumption as food products. Slaughterhouses which process meat not intended for human consumption are sometimes referred to as Knacker's yards or Knackeries. https://www.definitions.net/definition/slaughterhouse   Fish landing centres or sites are associated with small-scale marine and inland fisheries. They provide a location for first point of sale for products and provide a place where fishers can leave their boats and obtain supplies such as food, fuel and ice. The facilities, services and access to market vary. Source: https://www.fao.org/flw-in-fish-value-chains/value-chain/flw-in-fish-value-chainsvalue-chainwholesale/landing-sites-in-small-scale-fisheries/es/#:~:text=Fish%20landing%20centres%20or%20sites,as%20food%2C%20fuel%20and%20ice. |
|  | **Distribution infrastructure** | Roads | Road infrastructure is understood to include all physical assets within the road reserve, including not only the road itself, but all associated furniture (signage etc), and all earthworks, drainage, structures (culverts, bridges, buildings etc). Source: https://trid.trb.org/view/704839  Types of road • Murrum roads • Gravel roads • Earthen roads • Kankar roads  • Bitumious road • Concrete road https://theconstructor.org/transportation/classification-of-roads/17470/ |
|  |  | Railways | Railway infrastructure includes all the structures, buildings, land, and equipment to support rail lines. Railway infrastructure isn’t just limited to tracks; it includes all the structures, buildings, land, and equipment to support the rail lines. That support includes management, passenger, freight transport, and maintenance. Source: https://www.ferrovial.com/en/resources/railway-infrastructures/#:~:text=Railroad%20infrastructure%20includes%20all%20the,to%20support%20the%20rail%20lines.  Types of railways • Surface railways • Elevated railways  • Underground railways   https://theconstructor.org/transportation/types-railway-systems/554358/ |
|  |  | Bridges | A bridge is to allow people or cargo easy passage over an obstacle by providing a route that would otherwise be uneven or impossible. Source: https://www.lawinsider.com/dictionary/road-and-bridge-infrastructure-projects |
|  | **Information infrastructure** | Information centres (community info. centres, radios stations, information boards) | Information centre is a public space where community members have shared public access to ICT and use it to implement social development programs, support the social and personal development of the individuals, communities and contribute to improving the quality of life of community people (Heek, 2002)  The radio station subsystem (RSS) is the physical equipment that provides coverage to prescribed geographical areas, known as cells. It contains equipment required to communicate with the user equipment. Functionally, an RSS consists of a control function performed by the base station controller (BSC) and a transmitting/receiving function carried out by the base station transceiver (BTS) system. The BTS is the radio transmission/receiving equipment and covers a cell. An RSS can serve several cells and can have multiple base station transceivers (Garg, V., 2010). |
|  |  | Telecommunication masts | A telecommunication mast is a freestanding structure which supports antennas at a height where they can transmit and receive radio waves (Bello, 2010). |
| **Outcomes** |  |  |  |
|  | **Nutritious diets** | Food availability: farm level | Food production or yield (e.g. quantity of food produced per area (kg/hectare) |
|  |  | Food Availability: market level | **Availability of nutritious food**s (fruits, vegetables, dairy, eggs, meat, fish, legumes, nuts) (e.g. Specific metrics could be market volumes sold or transported or aggregated, number of market stands selling these items, etc.), **Market Level Dietary Score (MLDS)**: Number of foods or food groups available in local markets at a given point in time. |
|  |  | Food affordability | Volatility of food prices, change in income that leads to improve diet, household food expenditure, Food Insecurity Experience Scale (FIES), Household Food Insecurity Access Scale (HFIAS), Household Hunger Scale (HHS) |
|  |  | Food accessibility (physical) | Presence of food market, proximity, physical presence of food, food vendors |
|  |  | Food consumption and experiences | Food Consumption Score (FCS), Food Insecurity Experience Scale (FIES), Household Food Insecurity Access Scale (HFIAS), Household Hunger Scale (HHS) |
|  |  | Diet quality: individual dietary diversity level | Individual dietary diversity scores (DDS), Minimum Acceptable Diet (MAD) for infant/child (6 to 23 months), Minimum Dietary Diversity (MDD) for infant/child (6 to 23 months), Minimum Dietary Diversity for Women between 15 - 49 years (MDD-W), Mean adequacy ratio (MAR): measures an individual’s intake of nutrient, Food variety score, |
|  |  | Diet quality: household dietary diversity level | Household dietary diversity score, Food variety score, Food groups consumed, Food Consumption Score (FCS), dietary energy (kcal) etc |
|  |  | Sociocultural dimensions of foods | WENI is a nutrition-centred metric of empowerment that can be used to measure, track and identify barriers to nutritional empowerment. |
|  | **Women's economic empowerment** | ***The Five Domains of Empowerment (5DE)*** |  |
|  |  | *Agricultural production* | Sole or joint decision making over food and cash-crop farming, livestock, and fisheries as well as autonomy in agricultural production.  Input in production decisions, Autonomy in production |
|  |  | Access, control over productive resources | Ownership, access to, and decision making power over productive resources such as land, livestock, agricultural equipment, consumer durables, and credit  Ownership of assets, Purchase, sale or transfer of assets, Access to, and decisions about credit |
|  |  | Control over income | Sole or joint control over income and expenditures  Control over the use of income |
|  |  | Time | Allocation of time to productive and domestic tasks and satisfaction with the available time for leisure activities, workload and leisure. |
|  |  | Leadership | Membership in economic or social groups and comfort in speaking in public  Group member, speaking in public |
|  |  | Gender Parity Index (GPI) | The GPI is a relative inequality measure that reflects the inequality in 5DE profiles between the primary adult male and female in each household |
|  |  | Income/employment | Woman earns an income or employed, Income from agriculture, Income from non-farm employment |
|  |  | WEAI (pro-WEAI, A-WEAI) | An index based on a combination of one or more of the indicators: Agricultural production, access, control over productive resources; control over income; time; leadership; |
|  | **Gender equality** | Economic Opportunities and Outcomes | Women and men have equal opportunities in agricultural production systems, value chains, markets, resources (GAGP) and entrepreneurship |
|  |  | Social Outcomes | Discriminatory and unequal social, cultural and gender norms change to enable women and men to participate equally in household and community institutions |
|  |  | Leadership, Agency and Collective Action | Women’s agency, leadership and decision-making recognized and affirmed in the household and community. Women can engage in collective action to protect their interests |
|  |  | Reduced Exposure to Risk | Gender-based violence legislation and institutions exist to protect against GBV, and women are empowered to take action, leading to low and reduced rates of GBV |
| **Adverse effects** | **Nutritious diets** |  | **The study reports any adverse effects on nutritious diet** |
|  | **Women's economic empowerment** |  | **The study reports any adverse effects on women's economic empowerment** |
|  | **Gender equality** |  | **The study reports any adverse effects on Gender equality** |
| **Study Design** |  |  |  |
|  | **Experimental** |  | A study design that identify the treatment and control group through random assignment. The best example for this is RCT (Randomized Control Trials) |
|  | **Quasi experimental (including natural experiment)** |  | a quasi-experimental (natural experiment) design identifies a treatment and control group, but not through randomization. Common quasi-experimental (natural experimental) designs are difference in differences, regression discontinuity design, matching. |
|  | **Regression based study** |  | Regression based studies does not identify treatment and control groups but try to establish an effect using various regression approaches, including fixed effects, random effects, instrumental variable (two-stage least square), Heckman selection model, endogenous switching regression. |
|  | **Qualitative study** |  | In-depth interviews, Focus group discussion, key informant interviews, interviews etc, Inductive, deductive, exploratory qualitative studies, ethnography, grounded theory, |
|  | **Reviews (systematic, meta-analysis, scoping reviews)** |  | Systematic review, meta-analysis and scoping papers involving primary studies |
|  |  |  |  |
| **Publication status** | Published | Completed | Published in a scientific or academic journal |
|  | Unpublished | Ongoing | Institutional working papers, policy brief, evaluation reports, study protocols |
|  |  |  |  |
| **Publication type** | Peer review articles |  | Articles are written by experts and are reviewed by several other experts in the field before the article is published in the journal to ensure the article’s quality (source: https://www.angelo.edu/library/handouts/peerrev.php, accessed 25/08/2022) |
|  | Preprint-peer review article |  | Preprints" are preliminary versions of scientific manuscripts that researchers share by posting to online platforms known as preprint servers before peer-review and publication in an academic journal. (Source: https://library.cumc.columbia.edu/kb/what_is_preprint#:~:text=%22Preprints%22%20are%20preliminary%20versions%20of,preprints%20and%20their%20associated%20data. Accessed 25/08/2022) |
|  | Policy brief |  | A policy brief is a concise summary of a particular issue, the policy options to deal with it, and some recommendations on the best option. It is aimed at government policymakers and others who are interested in formulating or influencing policy. (Source: https://www.fao.org/3/i2195e/i2195e03.pdf, accessed 25/0/08/2022) |
|  | Report |  | Reports on performance of a programme or an intervention (source: https://www.oecd.org/derec/canada/35138852.pdf, accessed 25/08/2022) The systematic and objective assessment of an on-going or completed project, programme or policy, its design, implementation and results (Source: https://stats.oecd.org/glossary/detail.asp?ID=7097, accessed: 25/08/2022) |
|  | Working paper |  | Working Papers are pre-publication versions of academic articles, book chapters, or reviews (Source: https://www.princeton.edu/~pswpc/about/about.html accessed 24/08/2022.) |
|  | Conference paper |  | Paper presented at a conference (Source: https://writingcenter.unc.edu/tips-and-tools/conference-papers/ accessed 25/08/2022) |
|  | Book chapter |  |  |
| **Date of publication** | Select date |  | Date paper was published in a journal and not when it was indexed on-line |

**IINDWEGE SEARCH STRATEGY AND RIS DOWNLOADS REPORT**

**Appendix F:** **Databases used**

| **Name of database** | **Dates of coverage** | **Date of final search** |
| --- | --- | --- |
| PubMed | 07/06/2022 | 13/07/2022 |
| GreenFILE | 09/06/2022 | 22/08/2022 |
| Cab Direct | 12/06/2022 | 24/07/2022 |
